# SARS-CoV-2 Omicron variant: Antibody evasion and cryo-EM structure of spike protein–ACE2 complex

**DOI:** 10.1126/science.abn7760

**Published:** 2022-01-20

**Authors:** Dhiraj Mannar, James W. Saville, Xing Zhu, Shanti S. Srivastava, Alison M. Berezuk, Katharine S. Tuttle, Ana Citlali Marquez, Inna Sekirov, Sriram Subramaniam

**Affiliations:** ^1^ Department of Biochemistry and Molecular Biology, University of British Columbia, Vancouver, BC, Canada.; ^2^ BC Center for Disease Control Public Health Laboratory, Vancouver, BC, Canada.; ^3^ Department of Pathology and Laboratory Medicine, University of British Columbia, Vancouver, BC, Canada.; ^4^ Gandeeva Therapeutics, Inc., Vancouver, BC, Canada.

## Abstract

The newly reported Omicron variant is poised to replace Delta as the most prevalent severe acute respiratory syndrome coronavirus 2 (SARS-CoV-2) variant across the world. Cryo–electron microscopy (cryo-EM) structural analysis of the Omicron variant spike protein in complex with human angiotensin-converting enzyme 2 (ACE2) reveals new salt bridges and hydrogen bonds formed by mutated residues arginine-493, serine-496, and arginine-498 in the receptor binding domain with ACE2. These interactions appear to compensate for other Omicron mutations such as the substitution of asparagine for lysine at position 417 (K417N) that are known to reduce ACE2 binding affinity, resulting in similar biochemical ACE2 binding affinities for the Delta and Omicron variants. Neutralization assays show that pseudoviruses that display the Omicron spike protein exhibit increased antibody evasion. The increase in antibody evasion and the retention of strong interactions at the ACE2 interface thus represent important molecular features that likely contribute to the rapid spread of the Omicron variant.

## Antibody-evading Omicron keeps function

The Omicron variant of severe acute respiratory syndrome coronavirus 2 was reported in November 2021 and was quickly identified as a variant of concern because of its rapid spread. Relative to the original Wuhan-Hu-1 strain, this variant has 37 mutations in the spike protein that is responsible for binding and entry into host cells. Fifteen mutations are in the receptor-binding domain, which binds the host angiotensin-converting enzyme 2 (ACE2) receptor and is also the target of many neutralizing antibodies. Mannar *et al*. report a structure of the Omicron variant spike protein bound to human ACE2. The structure rationalizes the evasion of antibodies elicited by previous vaccination or infection and shows how mutations that weaken ACE2 binding are compensated for by mutations that make new interactions. —VV

## Teaser

Cryo-EM analysis of the Omicron spike protein reveals how ACE2 binding occurs despite high mutational escape from antibodies.

The Omicron (B.1.1.529) variant of severe acute respiratory syndrome coronavirus 2 (SARS-CoV-2), first reported in November 2021, was quickly identified as a variant of concern with the potential to spread rapidly across the world. This concern is heightened because the Omicron variant is now circulating even among doubly vaccinated individuals. SARS-CoV-2 relies on a trimeric spike protein for host cell entry via recognition of the angiotensin-converting enzyme 2 (ACE2) receptor. The Omicron variant spike protein has 37 mutations, as compared to 12 mutations in the Gamma variant spike protein, which was previously the variant with the greatest number of spike protein mutations (*1*). Understanding the consequences of these mutations for ACE2 receptor binding and neutralizing antibody evasion is important in guiding the development of effective therapeutics to limit the spread of the Omicron variant and related variants.

**Fig. 1.**
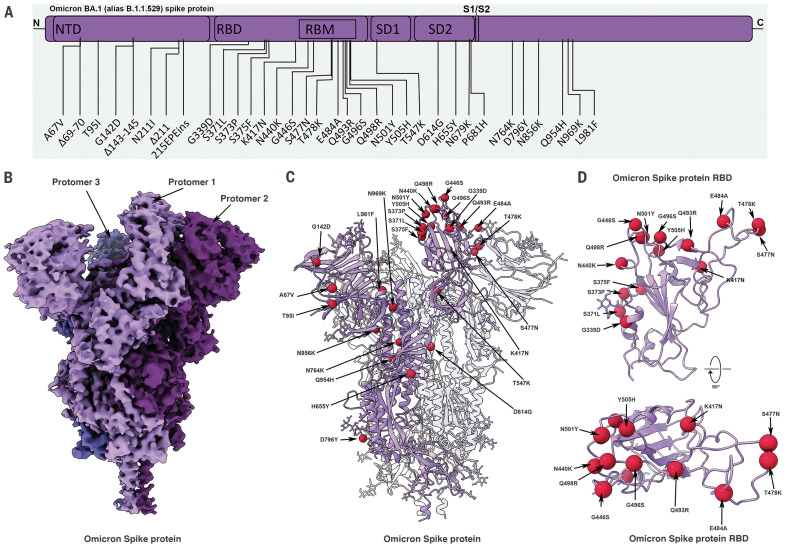
Cryo-EM structure of the Omicron spike protein. (**A**) A schematic diagram illustrating the domain arrangement of the spike protein. Mutations present in the Omicron variant spike protein are labeled. RBM, receptor binding motif. (**B**) Cryo-EM map of the Omicron spike protein at 2.79-Å resolution. Protomers are colored in different shades of purple. (**C**) Cryo-EM structure of Omicron spike protein indicating the locations of modeled mutations on one protomer. (**D**) The Omicron spike protein RBD shown in two orthogonal orientations with Cα positions of the mutated residues shown as red spheres. Single-letter abbreviations for the amino acid residues are as follows: A, Ala; C, Cys; D, Asp; E, Glu; F, Phe; G, Gly; H, His; I, Ile; K, Lys; L, Leu; M, Met; N, Asn; P, Pro; Q, Gln; R, Arg; S, Ser; T, Thr; V, Val; W, Trp; and Y, Tyr.

The spike protein comprises two domains: the S1 domain, which contains the receptor binding domain (RBD), and the S2 domain, which is responsible for membrane fusion. The Omicron variant has 37 mutations (Fig. 1A) in the spike protein relative to the initial Wuhan-Hu-1 strain, with 15 of them present in the RBD (*1*). The RBD mediates attachment to human cells through the ACE2 receptor and is the primary target of neutralizing antibodies (*2*, *3*). The Delta variant, which was the predominant SARS-CoV-2 lineage until the emergence of Omicron, has seven mutations in the spike protein relative to the Wuhan-Hu-1 strain, with two mutations falling within its RBD. Of the Delta spike protein mutations, two [T478K (Thr^478^→Lys) in the RBD and D614G (Asp^614^→Gly) at the C terminus of S1] are shared with the Omicron strain. Analysis of the sequence of the Omicron genome suggests that it is not derived from any of the variants circulating at present and may have a different origin (*4*).

Cryo–electron microscopy (cryo-EM) structural analysis of the Omicron spike protein ectodomain shows that the overall organization of the trimer is similar to that observed for the ancestral strain (*5*–*7*) and all earlier variants (*8*–*10*) (Fig. 1B and table S1). The RBD in one of the protomers (protomer 1) is well-resolved and is in the “down” position, whereas the other two RBDs are less well-resolved because they are flexible relative to the rest of the spike protein polypeptide. Similarly, the amino terminal domain (NTD) is poorly resolved, reflecting the dynamic and flexible nature of this domain. The mutations in the Omicron variant spike protein are distributed both on the surface and the interior of the spike protein (Fig. 1C), including the NTD and RBD regions. The mutations in the RBD are predominantly distributed on one face of the domain (Fig. 1D), which spans regions that bind ACE2 as well as those that form epitopes for numerous neutralizing antibodies (*11*).

**Fig. 2.**
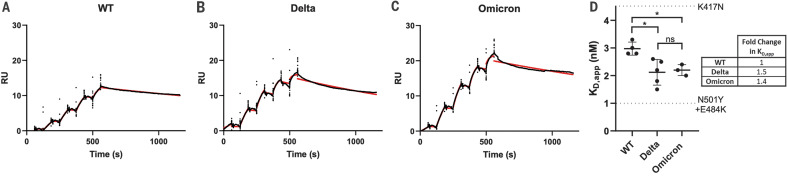
SPR analysis of the wild-type, Delta, and Omicron spike protein affinities for human ACE2. (**A** to **C**) Representative traces of single-cycle kinetic analyses of spike protein–ACE2 binding. The raw data (black) is fit (red) to a model using a 1:1 binding stoichiometry from which apparent dissociation constants were derived. The curves were obtained by injecting 6.25, 31.25, 62.5, 125, and 250 nM of each spike protein in successive cycles. RU, response units; WT, wild type. (**D**) Quantitation of apparent dissociation constants (*K*_D,app_) for the wild-type, Delta, and Omicron spike protein–ACE2 interactions. The standard deviation obtained from at least three technical replicates is shown. Horizontal dotted lines are plotted for mutants carrying only K417N (top) or N501Y and E484K (Glu^484^→Lys; bottom) mutations to demonstrate the range of this assay (see fig. S2 for binding data). A Tukey’s multiple comparisons test was performed on the wild-type, Delta, and Omicron binding affinities (*P ≤ 0.05; ns, not significant). A table highlighting the fold changes in *K*_D,app_ for the Delta and Omicron spike protein–ACE2 interactions relative to wild type is shown.

The Omicron variant shares RBD mutations with previous variants of concern [K417N (Lys^417^→Asn), T478K, and N501Y (Asn^501^→Tyr)]. The N501Y and K417N mutations impart increased and decreased ACE2 binding affinities, respectively (*8*, *12*–*16*). These mutational effects preserve the same general impact on ACE2 affinity when present in isolation or in combination with other RBD mutations (*12*). However, the Omicron RBD contains additional mutations, most of which have been shown to decrease receptor binding in a high-throughput assay (table S2) (*17*), with the exception of G339D (Gly^339^→Asp), N440K (Asn^440^→Lys), S447N (Ser^447^→Asn), and Q498R (Gln^498^→Arg) (*17*, *18*). To measure the impact of Omicron spike protein mutations on human ACE2 binding affinity, we performed surface plasmon resonance (SPR) studies and compared the resulting apparent binding affinities (*K*_D,app_) to wild-type and Delta spike proteins (Fig. 2). “Wild type” is used in this work to refer to the ancestral Wuhan-Hu-1 strain with the addition of the D614G mutation. Although the Omicron spike protein exhibits a measurable increase in apparent affinity for ACE2 relative to the wild-type spike protein [in agreement with a recent preprint (*19*)], the apparent ACE2 affinity is similar for both the Delta and Omicron variants (Fig. 2D). Despite harboring several RBD mutations that decrease ACE2 binding (fig. S2) (*12*, *16*, *17*), the preservation of overall ACE2 binding affinity for the Omicron spike protein suggests there are compensatory mutations that restore higher affinity for ACE2. Such mutational effects should be possible to visualize in a high-resolution structure of the spike protein–ACE2 complex.

**Fig. 3.**
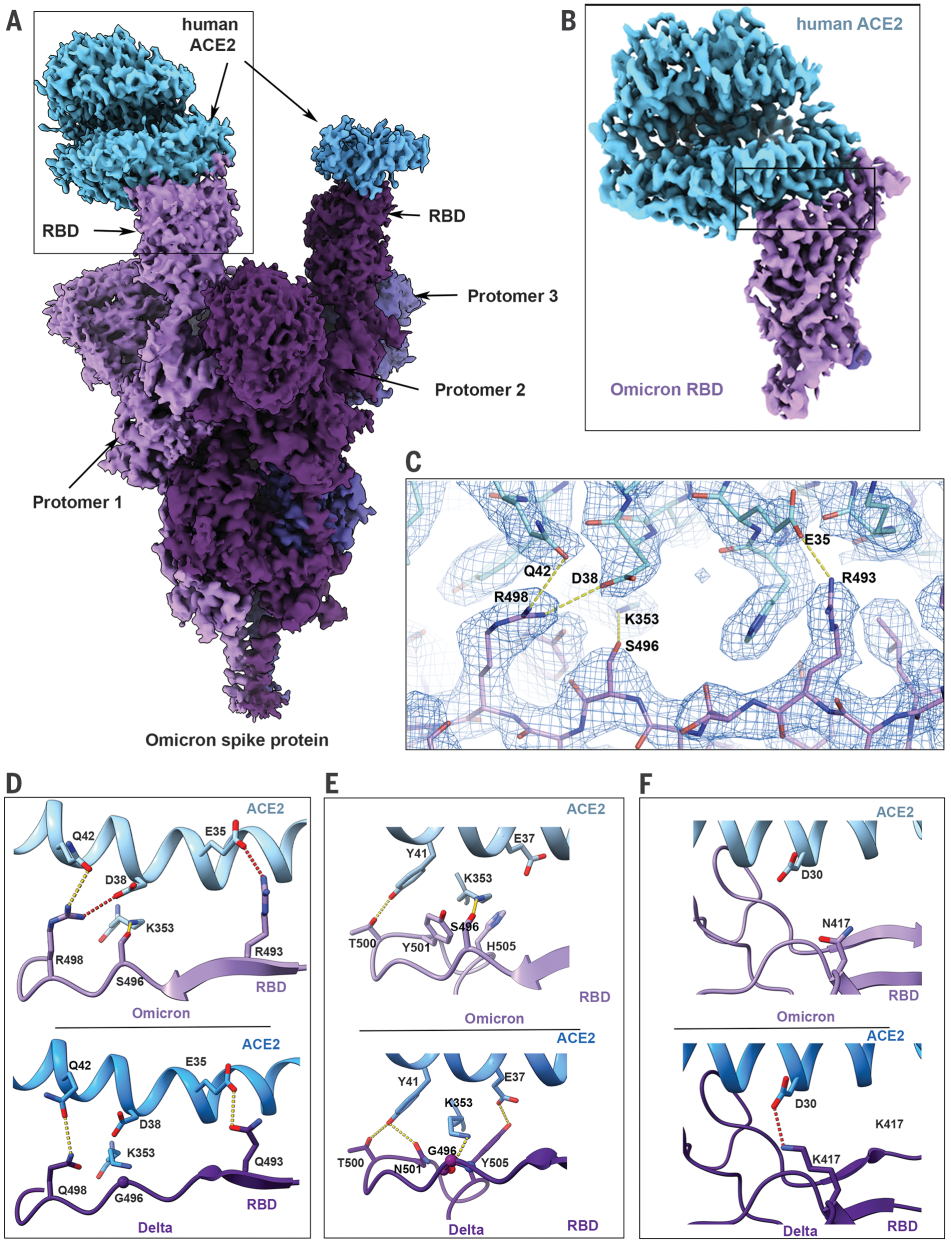
Cryo-EM structure of the Omicron spike protein–ACE2 complex. (**A**) Cryo-EM map of the Omicron spike protein in complex with human ACE2 at 2.45-Å resolution after global refinement. The three protomers are colored in different shades of purple, and the density for bound ACE2 is colored in blue. (**B**) Cryo-EM map of the Omicron spike protein RBD in complex with ACE2 at 2.66-Å resolution after focused refinement. The boxed area indicates the region highlighted in (C). (**C**) Cryo-EM density mesh at the Omicron spike protein RBD–ACE2 interface, with fitted atomic model. Yellow and red dashed lines represent new hydrogen bonds and ionic interactions, respectively. (**D** to **F**) Comparison of the RBD-ACE2 interface between the Omicron (top) and Delta (bottom) variants. Compared with the Delta variant, new interactions are formed as a result of the mutations Q493R, G496S, and Q498R (D) and local structural changes owing to the N501Y and Y505H (Tyr^505^→His) mutations (E) present in the Omicron variant. The salt bridge between Delta RBD K417 and ACE2 D30 that is present in the Delta variant spike protein but lost in the Omicron variant is highlighted in (F). Yellow and red dashed lines represent hydrogen bonds and ionic interactions, respectively.

Cryo-EM structural analysis of the human ACE2–Omicron spike protein complex shows strong density for ACE2 bound to the RBD of one of the protomers in the “up” position (Fig. 3A and table S1). Weaker density is observed for a second bound ACE2, suggesting partial occupancy of a second RBD under our experimental conditions. We focus on the structure of the ACE2–spike protein interface in the most strongly bound ACE2 molecule. Focused refinement of the RBD-ACE2 region resulted in a density map with a resolution of 2.66 Å at the spike protein–ACE2 interface (Fig. 3B), allowing the visualization of side chains involved in the interface (Fig. 3C). In Fig. 3, D to F, we compare the key interactions at this interface in the Omicron variant with corresponding interactions that we have recently reported for the Delta variant (*20*). In the Delta variant–ACE2 complex, there are hydrogen bonds formed by residues Q493 and Q498 on the spike protein with residues E35 (E, Glu) and Q42, respectively, on ACE2 (Fig. 3D). In the Omicron variant, three mutations are observed in this stretch: Q493R (Gln^493^→Arg), G496S (Gly^496^→Ser), and Q498R. Residue R493 replaces the hydrogen bond to ACE2 residue E35 with a new salt bridge, whereas residue R498 forms a new salt bridge with ACE2 residue D38 while maintaining a hydrogen bond interaction with ACE2 residue Q42. RBD residue S496 adds a new interaction at the interface by forming a hydrogen bond with ACE2 residue K353 (Fig. 3D). Additionally, the mutated residue Y501 in the Omicron RBD makes π-stacking interactions with Y41 in ACE2, as previously seen in the Alpha (B.1.1.7), Beta (B.1.351), and Gamma (P.1) variants (*8*, *12*), whereas mutated residue H505 (H, His) is not hydrogen-bonded to E37 in ACE2, in contrast to what we reported previously for the Y505 residue (Fig. 3E) (*20*).

These new interactions are offset by the loss of a key salt bridge between spike protein residue K417 and ACE2 residue D30 that is present in the Delta variant (Fig. 3F). In isolation, the K417N mutant displays reduced ACE2 binding affinity (*12*, *16*), but our findings suggest that the new mutations in the Omicron interface have a compensatory effect on the strength of ACE2 binding, providing an explanation for the similar ACE2 binding affinities that are observed (Fig. 2).

We next investigated the effects of Omicron mutations on neutralization by (i) a selection of monoclonal antibodies, (ii) sera obtained from 30 doubly vaccinated individuals with no prior history of COVID-19 infection, and (iii) sera obtained from a set of 68 unvaccinated convalescent patients who recovered from infection with either the Alpha, Gamma, or Delta variants. (A summary of patient demographics is in table S3.) We performed neutralization experiments using pseudoviruses that incorporate the wild-type, Delta variant, or Omicron variant spike proteins and compared the ability of these pseudovirions to evade antibodies. We compare evasion relative to the Delta variant, given that the Omicron variant is rapidly supplanting the Delta variant in global prevalence, and to wild-type SARS-CoV-2, given that most SARS-CoV-2 vaccine immunogens at this time are based on this sequence (*21*).

**Fig. 4.**
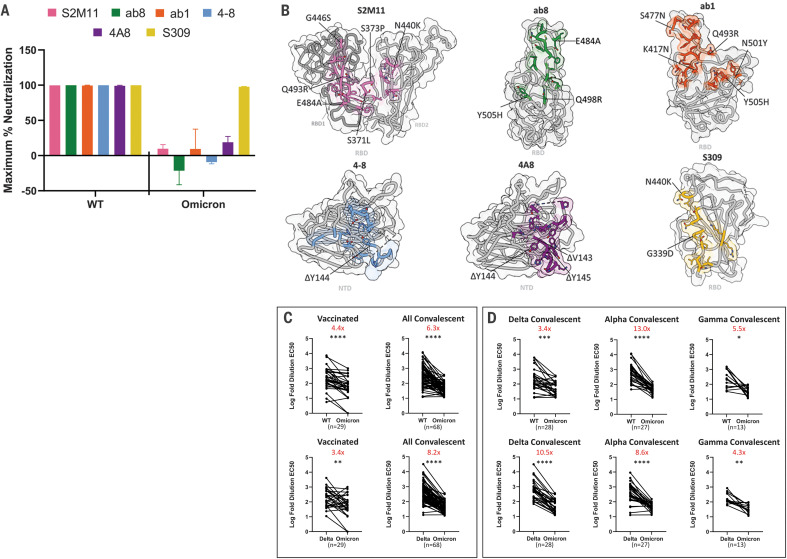
Monoclonal antibodies and vaccinated and convalescent patient-derived sera exhibit decreased Omicron neutralization potency. (**A**) Maximum neutralization achieved by the indicated monoclonal antibodies against wild-type and Omicron pseudoviruses (*n* = 3 technical replicates). Error bars denote standard deviation of the mean. (**B**) Antibody binding footprints for the monoclonal antibodies tested in this study. Omicron spike protein mutations that fall within each antibody footprint are labeled. (**C**) Log-fold 50% effective concentration (EC50) dilutions for vaccinated and convalescent patient sera for either wild-type (WT) versus Omicron variant pseudoviruses (top) or Delta versus Omicron variant pseudoviruses (bottom). (**D**) As in (C) but with a breakdown of the convalescent patients into previous infection with Delta, Alpha, or Gamma variants of concern. A pairwise statistical significance test was performed using the Wilcoxon matched pairs test (*P ≤ 0.05; **P ≤ 0.01; ***P ≤ 0.001; ****P ≤ 0.0001). The fold change in the geometric mean between the two groups is shown in red at the top of each plot.

We used a panel of neutralizing monoclonal antibodies that include four RBD-directed antibodies [ab1, ab8, S309, and S2M11; (*22*–*25*)] and two NTD-directed antibodies [4-8 and 4A8; (*26*, *27*)] to investigate the impact of Omicron RBD and NTD mutations on monoclonal antibody escape. In contrast to wild-type SARS-CoV-2 and the Alpha (B.1.1.7), Gamma (P.1), Kappa (B.1.617.1), and Delta (B.1.617.2) variants, the Omicron variant could not be completely neutralized at maximum concentrations of five of the six antibodies tested (Fig. 4A and fig. S4) (*20*, *28*). The loss of neutralizing activity for both the NTD-directed antibodies (4-8 and 4A8) against Omicron is likely due to the Δ144-145 deletion, which falls within the footprint of both of these antibodies (Fig. 4B). The escape from RBD-directed antibodies S2M11, ab8, and ab1 is likely due to the numerous Omicron mutations that lie within their respective footprints (Fig. 4B). By contrast, S309 (an antibody undergoing evaluation in clinical trials for treating patients with COVID-19) was able to fully neutralize the Omicron variant, consistent with previous reports that show retained neutralization capacity of S309 despite a mild decrease in potency (*19*, *29*–*31*). The unusually high number of mutations in the Omicron variant spike protein thus appear to confer broad antibody escape relative to previously emerged variants of SARS-CoV-2, consistent with emerging reports (*19*).

Sera obtained from patients not exposed to SARS-CoV-2 (prepandemic) showed negligible neutralization activity against wild-type SARS-CoV-2 and both the Delta and Omicron variants (fig. S5). Sera from either vaccinated or convalescent patients exhibited potent neutralization of wild-type pseudoviruses (figs. S6 to S9); sera from convalescent patients displayed, on average, a 6.3× decrease in ability to neutralize the Omicron variant relative to wild type (Fig. 4C, top). Sera from the vaccinated cohort also displayed reduced neutralization ability (4.4× decrease on average) with a wider variation driven by some individuals that showed greater loss of neutralization ability against Omicron. The comparison of change in neutralization potential between the Delta and Omicron variants is perhaps more relevant given the previous worldwide dominance of the Delta variant. Sera from convalescent patients shows an even greater drop in neutralization potency relative to the Delta variant (8.2× decrease), whereas the vaccinated group also shows reduction in potency, although to a lesser extent (3.4× decrease) (Fig. 4C, bottom).

A finer analysis of the unvaccinated convalescent cohort stratified into those who recovered from infection with either the Delta, Alpha, or Gamma variants (Fig. 4D) highlights the reduction in neutralization potency against the Omicron variant relative to the Delta variant in all populations, with especially notable drops for patients who recovered from infection with the earlier Alpha and Delta variants. The findings we report here are consistent with several other recent reports (*19*, *32*–*34*) that support the finding that the Omicron variant is more resistant to neutralization dependent on prior infection with an earlier variant or vaccination than any other variant of concern that has emerged over the course of the COVID-19 pandemic.

The large number of mutations on the surface of the spike protein, including the immunodominant RBD (Fig. 1), would be expected to help the virus escape antibodies elicited by vaccination or prior infection. It is interesting that the Omicron variant evolved to retain its ability to bind ACE2 efficiently despite these extensive mutations. The cryo-EM structure of the spike protein–ACE2 complex provides a structural rationale for how this is achieved: Interactions involving the new mutations in the Omicron variant at residues 493, 496, 498, and 501 appear to restore ACE2 binding efficiency that would be lost as a result of other mutations such as K417N. The Omicron variant thus appears to have evolved to selectively balance an increase in escape from neutralization with its ability to interact efficiently with ACE2. The increase in antibody evasion and the retention of strong interactions at the ACE2 interface are thus factors that likely contribute to the increase in transmissibility of the Omicron variant.

## Supplementary Material

20220120-1

## Supplementary Materials

### This PDF file includes:

Materials and Methods Figs. S1 to S9 Tables S1 to S3 References (*35*–*39*)

### Other Supplementary Material for this manuscript includes the following:

MDAR Reproducibility Checklist

## Notes

View/request a protocol for this paper from *Bio-protocol*.
