# Supplementary material for: SARS-CoV-2 Omicron variant: Antibody evasion and cryo-EM structure of spike protein–ACE2 complex
Source: Science. 2022 Jan 20;375(6582):760–4. doi: 10.1126/science.abn7760 (PMC9799367; doi:10.1126/science.abn7760)
Supplement: 20220120-1 [file science.abn7760.v1.pdf]

Science

REPORTS

Cite as: D. Mannar *et al.*, *Science* 10.1126/science.abn7760 (2022).

SARS-CoV-2 Omicron variant: Antibody evasion and cryo-EM structure of spike protein–ACE2 complex

Dhiraj Mannar[1]†, James W. Saville[1]†, Xing Zhu[1]†, Shanti S. Srivastava[1], Alison M. Berezuk[1], Katharine S. Tuttle[1], Ana Citlali Marquez[2], Inna Sekirov[2,3], Sriram Subramaniam[1,4]*

[1]Department of Biochemistry and Molecular Biology, University of British Columbia, Vancouver, BC, Canada. [2]BC Center for Disease Control Public Health Laboratory, Vancouver, BC, Canada. [3]Department of Pathology and Laboratory Medicine, University of British Columbia, Vancouver, BC, Canada. [4]Gandeeva Therapeutics, Inc., Vancouver, BC, Canada.

†These authors contributed equally to this work. *Corresponding author. Email: sriram.subramaniam@ubc.ca

The newly reported Omicron variant is poised to replace Delta as the most prevalent SARS-CoV-2 variant across the world. Cryo-EM structural analysis of the Omicron variant spike protein in complex with human ACE2 reveals new salt bridges and hydrogen bonds formed by mutated residues R493, S496 and R498 in the RBD with ACE2. These interactions appear to compensate for other Omicron mutations such as K417N known to reduce ACE2 binding affinity, resulting in similar biochemical ACE2 binding affinities for Delta and Omicron variants. Neutralization assays show that pseudoviruses displaying the Omicron spike protein exhibit increased antibody evasion. The increase in antibody evasion, together with retention of strong interactions at the ACE2 interface, thus represent important molecular features that likely contribute to the rapid spread of the Omicron variant.

The Omicron (B.1.1.529) variant of SARS-CoV-2, first reported in November 2021, was quickly identified as a variant of concern with potential to spread rapidly across the world. This concern is heightened as the Omicron variant is currently circulating even amongst doubly vaccinated individuals. SARS-CoV-2 relies on a trimeric spike protein for host cell entry via recognition of the angiotensin converting enzyme 2 (ACE2) receptor. The Omicron variant spike protein has 37 mutations as compared to 12 mutations in the Gamma variant, the most mutated variant to emerge before Omicron (*1*). Understanding the consequences of these mutations for ACE2 receptor binding and neutralizing antibody evasion is important in guiding the development of effective therapeutics to limit the spread of the Omicron and related variants.

The spike protein comprises 2 domains, the S1 domain, which contains the receptor binding domain (RBD) and the S2 domain responsible for membrane fusion. The Omicron variant has 37 mutations (Fig. 1A) in the spike protein relative to the initial Wuhan-Hu-1 strain, with 15 of them present in the receptor binding domain (RBD) (*1*). The RBD mediates attachment to human cells through the ACE2 receptor and is the primary target of neutralizing antibodies (*2*, *3*). The Delta variant, which was the predominant SARS-CoV-2 lineage until the emergence of Omicron, has 7 mutations in the spike protein relative to the Wuhan-Hu-1 strain, with 2 mutations falling within its RBD. Of the Delta spike mutations, two (T478K in the RBD and D614G at the C terminus of S1) are shared with the Omicron strain. Analysis of the sequence of the Omicron genome suggests that it is not derived from any of the currently circulating variants, and may have a different origin (*4*).

Cryo-EM structural analysis of the Omicron spike protein ectodomain shows that the overall organization of the trimer is similar to that observed for the ancestral strain (*5*–*7*) and all earlier variants (*8*–*10*) (Fig. 1B and table S1). The RBD in one of the protomers (protomer 1) is well-resolved and is in the “down” position, while the other two RBDs are less well-resolved because they are flexible relative to the rest of the spike protein polypeptide. Similarly, the amino terminal domain (NTD) is poorly resolved, reflecting the dynamic and flexible nature of this domain. The mutations in the Omicron variant spike protein are distributed both on the surface and the interior of the spike protein (Fig. 1C), including NTD and RBD regions. The mutations in the RBD are predominantly distributed on one face of the domain (Fig. 1D), which spans regions that bind ACE2 as well as those that form epitopes for numerous neutralizing antibodies (*11*).

The Omicron variant shares RBD mutations in common with previous variants of concern (K417N, T478K, and N501Y). The N501Y and K417N mutations impart increased and decreased ACE2 binding affinities respectively (*8*, *12*–*16*). These mutational effects preserve the same general impact on ACE2 affinity when present in isolation or in combination with other RBD mutations (*12*). However, the Omicron RBD contains additional mutations, the majority of which have been shown to decrease receptor binding in a high-throughput assay (table S2) (*17*), with the exception of G339D, N440K, S447N, and Q498R (*17*, *18*). To measure the impact of Omicron spike protein mutations on human ACE2 binding affinity, we performed surface plasmon resonance studies and compared the resulting apparent binding affinities ($K_{D,app}$) to

wild-type and Delta spikes (Fig. 2). Wild-type (WT) is used in this work to refer to the ancestral Wuhan-Hu-1 strain with the addition of the D614G mutation. While the Omicron spike protein exhibits a measurable increase in apparent affinity for ACE2 relative to the wild-type spike [in agreement with a recent preprint (*19*)], the apparent ACE2 affinity is similar for Delta and Omicron variants (Fig. 2D). Despite harboring several RBD mutations which decrease ACE2 binding (fig. S2) (*12*, *16*, *17*), the preservation of overall ACE2 binding affinity for the Omicron spike protein suggests there are compensatory mutations that restore higher affinity for ACE2. Such mutational effects should be possible to visualize in a high-resolution structure of the spike protein-ACE2 complex.

Cryo-EM structural analysis of the human ACE2-Omicron spike protein complex shows strong density for ACE2 bound to the RBD of one of the protomers in the "up" position (Fig. 3A and table S1). Weaker density is observed for a second bound ACE2, suggesting partial occupancy of a second RBD under our experimental conditions. We focus on the structure of the ACE2-spike protein interface in the most strongly bound ACE2 molecule. Focused refinement of the RBD-ACE2 region resulted in a density map with a resolution of 2.66 Å at the spike protein-ACE2 interface (Fig. 3B), allowing visualization of sidechains involved in the interface (Fig. 3C). In Fig. 3, D to F, we compare the key interactions at this interface in the Omicron variant with corresponding interactions that we have reported recently for the Delta variant (*20*). In the Delta variant-ACE2 complex, there are hydrogen bonds formed by residues Q493 and Q498 on the spike protein with residues E35 and Q42, respectively, on ACE2 (Fig. 3D). In the Omicron variant, three mutations are observed in this stretch: Q493R, G496S and Q498R. Residue R493 replaces the hydrogen bond to ACE2 residue E35 with a new salt bridge, while residue R498 forms a new salt bridge with ACE2 residue D38 while maintaining a hydrogen bond interaction with ACE2 residue Q42. RBD residue S496 adds a new interaction at the interface by forming a hydrogen bond with ACE2 residue K353 (Fig. 3D). Additionally, the mutated residue Y501 in the Omicron RBD makes pi stacking interactions with Y41 in ACE2 as previously seen in the Alpha (B.1.1.7), Beta (B.1.351), and Gamma (P.1) variants (*8*, *12*), while mutated residue H505 is not hydrogen-bonded to E37 in ACE2 in contrast to what we reported previously for the Y505 residue (Fig. 3E) (*20*).

These new interactions are offset by the loss of a key salt bridge between spike protein residue K417 and ACE2 residue D30 that is present in the Delta variant (Fig. 3F). In isolation, the K417N mutant displays reduced ACE2 binding affinity (*12*, *16*), but our findings suggest that the new mutations in the Omicron interface have a compensatory effect on the strength of ACE2 binding, providing an explanation for the similar ACE2 binding affinities observed (Fig. 2).

We next investigated the effects of Omicron mutations on neutralization by (i) a selection of monoclonal antibodies, (ii) sera obtained from 30 doubly vaccinated individuals with no prior history of COVID-19 infection and (iii) sera obtained from a set of 68 unvaccinated convalescent patients who recovered from infection by either Alpha, Gamma or Delta variants (summary of patient demographics is in table S3). We performed neutralization experiments using pseudoviruses that incorporate the wild-type, Delta, or Omicron variant S proteins and compared the ability of these pseudovirions to evade antibodies. We compare evasion relative to the Delta variant given that the Omicron variant is rapidly supplanting the Delta variant in global prevalence, and to wild-type SARS-CoV-2 given that the majority of current SARS-CoV-2 vaccine immunogens are based on this sequence (*21*).

We used a panel of neutralizing monoclonal antibodies that include RBD-directed antibodies [ab1, ab8, S309, S2M11; (*22*–*25*)] and two NTD-directed antibodies [4-8 and 4A8; (*26*, *27*)] to investigate the impact of Omicron RBD and NTD mutations on monoclonal antibody escape. In contrast to the wild-type, Alpha (B.1.1.7), Gamma (P.1), Kappa (B.1.617.1), and Delta (B.1.617.2) variants of SARS-CoV-2, the Omicron variant could not be completely neutralized at maximum concentrations of five of the six antibodies tested (Fig. 4A and fig. S4) (*20*, *28*). The loss of neutralizing activity for both the NTD-directed antibodies (4-8 and 4A8) against Omicron is likely due to the Δ144-145 deletion which falls within the footprint of both of these antibodies (Fig. 4B). The escape from RBD directed antibodies S2M11, ab8 and ab1 is likely due to the numerous Omicron mutations which lie within their respective footprints (Fig. 4B). In contrast, S309 (an antibody undergoing evaluation in clinical trials for treating patients with COVID-19) was able to fully neutralize the Omicron variant, consistent with previous reports showing retained neutralization capacity of S309 despite a mild decrease in potency (*19*, *29*–*31*). The unusually high number of mutations in the Omicron variant spike protein thus appear to confer broad antibody escape relative to previously emerged variants of SARS-CoV-2, consistent with emerging reports (*19*).

Sera obtained from patients not exposed to SARS-CoV-2 (pre-pandemic) showed negligible neutralization activity against wild-type SARS-CoV-2 and both Delta and Omicron variants (fig. S5). Sera from either vaccinated or convalescent patients exhibited potent neutralization of wild-type pseudoviruses (figs. S6 to S9); sera from convalescent patients displayed on average a 6.3x decrease in ability to neutralize the Omicron variant relative to wild-type (Fig. 4C, top). Sera from the vaccinated cohort also displayed reduced neutralization ability (4.4x decrease on average) with a wider variation driven by some individuals that showed greater loss of neutralization ability against Omicron. The comparison of change in neutralization potential between Delta and

Omicron variants is perhaps more relevant given the previous world-wide dominance of the Delta variant. Sera from convalescent patients shows an even greater drop in neutralization potency relative to the Delta variant (8.2x decrease) while the vaccinated group also shows reduction in potency, although to a lesser extent (3.4x decrease) (Fig. 4C, bottom).

A finer analysis of the unvaccinated convalescent cohort stratified into those who recovered from infection by either Delta, Alpha, or Gamma variants (Fig. 4D) highlights the reduction in neutralization potency against the Omicron variant relative to the Delta variant in all populations, with especially striking drops for patients who recovered from infection from the earlier Alpha and Delta variants. The findings we report here are consistent with several other recent reports (*19*, *32–34*) supporting the finding that the Omicron variant is more resistant to neutralization dependent on prior infection with an earlier variant or vaccination than any other variant of concern that has emerged over the course of the COVID-19 pandemic.

The large number of mutations on the surface of the spike protein including the immunodominant RBD (Fig. 1) would be expected to help the virus escape antibodies elicited by vaccination or prior infection. It is interesting that the Omicron variant evolved to retain its ability to bind ACE2 efficiently despite these extensive mutations. The cryo-EM structure of the spike protein-ACE2 complex provides a structural rationale for how this is achieved: interactions involving the new mutations in the Omicron variant at residues 493, 496, 498, and 501 appear to restore ACE2 binding efficiency that would be lost due to other mutations such as K417N. The Omicron variant thus appears to have evolved to selectively balance an increase in escape from neutralization with its ability to interact efficiently with ACE2. The increase in antibody evasion and the retention of strong interactions at the ACE2 interface are thus factors that likely contribute to the increase in transmissibility of the Omicron variant.

REFERENCES AND NOTES

1. S. Elbe, G. Buckland-Merrett, Data, disease and diplomacy: GISAID's innovative contribution to global health. *Glob. Chall.* **1**, 33–46 (2017). doi:10.1002/gch2.1018 Medline
2. M. Hoffmann, H. Kleine-Weber, S. Schroeder, N. Krüger, T. Herrler, S. Erichsen, T. S. Schiergens, G. Herrler, N.-H. Wu, A. Nitsche, M. A. Müller, C. Drosten, S. Pöhlmann, SARS-CoV-2 cell entry depends on ACE2 and TMPRSS2 and is blocked by a clinically proven protease inhibitor. *Cell* **181**, 271–280.e8 (2020). doi:10.1016/j.cell.2020.02.052 Medline
3. L. Piccoli, Y.-J. Park, M. A. Tortorici, N. Czudnochowski, A. C. Walls, M. Beltramello, C. Silacci-Fregni, D. Pinto, L. E. Rosen, J. E. Bowen, O. J. Acton, S. Jaconi, B. Guarino, A. Minola, F. Zatta, N. Sprugasci, J. Bassi, A. Peter, A. De Marco, J. C. Nix, F. Mele, S. Jovic, B. F. Rodriguez, S. V. Gupta, F. Jin, G. Piumatti, G. Lo Presti, A. F. Pellanda, M. Biggiogero, M. Tarkowski, M. S. Pizzuto, E. Cameroni, C. Havenar-Daughton, M. Smithey, D. Hong, V. Lepori, E. Albanese, A. Ceschi, E. Bernasconi, L. Elzi, P. Ferrari, C. Garzoni, A. Riva, G. Snell, F. Sallusto, K. Fink, H. W. Virgin, A. Lanzavecchia, D. Corti, D. Veesler, Mapping neutralizing and immunodominant sites on the SARS-CoV-2 spike receptor-binding domain by structure-guided high-resolution serology. *Cell* **183**, 1024–1042.e21 (2020). doi:10.1016/j.cell.2020.09.037 Medline
4. C. Wei, K.-J. Shan, W. Wang, S. Zhang, Q. Huan, W. Qian, Evidence for a mouse origin of the SARS-CoV-2 Omicron variant. *J. Genet. Genomics* 10.1016/j.jgg.2021.12.003 (2021). doi:10.1016/j.jgg.2021.12.003 Medline
5. A. C. Walls, Y.-J. Park, M. A. Tortorici, A. Wall, A. T. McGuire, D. Veesler, Structure, function, and antigenicity of the SARS-CoV-2 spike glycoprotein. *Cell* **181**, 281–292.e6 (2020). doi:10.1016/j.cell.2020.02.058 Medline
6. D. Wrapp, N. Wang, K. S. Corbett, J. A. Goldsmith, C.-L. Hsieh, O. Abiona, B. S. Graham, J. S. McLellan, Cryo-EM structure of the 2019-nCoV spike in the prefusion conformation. *Science* **367**, 1260–1263 (2020). doi:10.1126/science.abb2507 Medline
7. J. Zhang, Y. Cai, T. Xiao, J. Lu, H. Peng, S. M. Sterling, R. M. Walsh Jr., S. Rits-Volloch, H. Zhu, A. N. Woosley, W. Yang, P. Sliz, B. Chen, Structural impact on SARS-CoV-2 spike protein by D614G substitution. *Science* **372**, 525–530 (2021). doi:10.1126/science.abf2303 Medline
8. X. Zhu, D. Mannar, S. S. Srivastava, A. M. Berezuk, J.-P. Demers, J. W. Saville, K. Leopold, W. Li, D. S. Dimitrov, K. S. Tuttle, S. Zhou, S. Chittori, S. Subramaniam, Cryo-electron microscopy structures of the N501Y SARS-CoV-2 spike protein in complex with ACE2 and 2 potent neutralizing antibodies. *PLOS Biol.* **19**, e3001237 (2021). doi:10.1371/journal.pbio.3001237 Medline
9. J. Zhang, T. Xiao, Y. Cai, C. L. Lavine, H. Peng, H. Zhu, K. Anand, P. Tong, A. Gautam, M. L. Mayer, R. M. Walsh Jr., S. Rits-Volloch, D. R. Wesemann, W. Yang, M. S. Seaman, J. Lu, B. Chen, Membrane fusion and immune evasion by the spike protein of SARS-CoV-2 Delta variant. *Science* **374**, 1353–1360 (2021). doi:10.1126/science.abl9463 Medline
10. S. M.-C. Gobeil, K. Janowska, S. McDowell, K. Mansouri, R. Parks, V. Stalls, M. F. Kopp, K. Manne, D. Li, K. Wiehe, K. O. Saunders, R. J. Edwards, B. Korber, B. F. Haynes, R. Henderson, P. Acharya, Effect of natural mutations of SARS-CoV-2 on spike structure, conformation, and antigenicity. *Science* **373**, eabi6226 (2021). doi:10.1126/science.abi6226 Medline
11. K. M. Hastie, H. Li, D. Bedinger, S. L. Schendel, S. M. Dennison, K. Li, V. Rayaprolu, X. Yu, C. Mann, M. Zandonatti, R. Diaz Avalos, D. Zyla, T. Buck, S. Hui, K. Shaffer, C. Hariharan, J. Yin, E. Olmedillas, A. Enriquez, D. Parekh, M. Abraha, E. Feeney, G. Q. Horn, Y. Aldon, H. Ali, S. Aracic, R. R. Cobb, R. S. Federman, J. M. Fernandez, J. Glanville, R. Green, G. Grigoryan, A. G. Lujan Hernandez, D. D. Ho, K. A. Huang, J. Ingraham, W. Jiang, P. Kellam, C. Kim, M. Kim, H. M. Kim, C. Kong, S. J. Krebs, F. Lan, G. Lang, S. Lee, C. L. Leung, J. Liu, Y. Lu, A. MacCamy, A. T. McGuire, A. L. Palser, T. H. Rabbitts, Z. Rikhtegaran Tehrani, M. M. Sajadi, R. W. Sanders, A. K. Sato, L. Schweizer, J. Seo, B. Shen, J. L. Snitselaar, L. Stamatatos, Y. Tan, M. T. Tomic, M. J. van Gils, S. Youssef, J. Yu, T. Z. Yuan, Q. Zhang, B. Peters, G. D. Tomaras, T. Germann, E. O. Saphire, CoVIC-DB team1, Defining variant-resistant epitopes targeted by SARS-CoV-2 antibodies: A global consortium study. *Science* **374**, 472–478 (2021). doi:10.1126/science.abh2315 Medline
12. D. Mannar, J. W. Saville, X. Zhu, S. S. Srivastava, A. M. Berezuk, S. Zhou, K. S. Tuttle, A. Kim, W. Li, D. S. Dimitrov, S. Subramaniam, Structural analysis of receptor binding domain mutations in SARS-CoV-2 variants of concern that modulate ACE2 and antibody binding. *Cell Rep.* **37**, 110156 (2021). doi:10.1016/j.celrep.2021.110156 Medline
13. M. I. Barton, S. A. MacGowan, M. A. Kutuzov, O. Dushek, G. J. Barton, P. A. van der Merwe, Effects of common mutations in the SARS-CoV-2 Spike RBD and its ligand, the human ACE2 receptor on binding affinity and kinetics. *eLife* **10**, e70658 (2021). doi:10.7554/eLife.70658 Medline
14. H. Liu, Q. Zhang, P. Wei, Z. Chen, K. Aviszus, J. Yang, W. Downing, C. Jiang, B. Liang, L. Reynoso, G. P. Downey, S. K. Frankel, J. Kappler, P. Marrack, G. Zhang, The basis of a more contagious 501Y.V1 variant of SARS-CoV-2. *Cell Res.* **31**, 720–722 (2021). doi:10.1038/s41422-021-00496-8 Medline
15. F. Tian, B. Tong, L. Sun, S. Shi, B. Zheng, Z. Wang, X. Dong, P. Zheng, N501Y mutation of spike protein in SARS-CoV-2 strengthens its binding to receptor ACE2. *eLife* **10**, e69091 (2021). doi:10.7554/eLife.69091 Medline
16. C. Laffeber, K. de Koning, R. Kanaar, J. H. G. Lebbink, Experimental evidence for enhanced receptor binding by rapidly spreading SARS-CoV-2 variants. *J. Mol. Biol.* **433**, 167058 (2021). doi:10.1016/j.jmb.2021.167058 Medline
17. T. N. Starr, A. J. Greaney, S. K. Hilton, D. Ellis, K. H. D. Crawford, A. S. Dingens, M. J. Navarro, J. E. Bowen, M. A. Tortorici, A. C. Walls, N. P. King, D. Veesler, J. D. Bloom, Deep mutational scanning of SARS-CoV-2 receptor binding domain reveals constraints on folding and ACE2 binding. *Cell* **182**, 1295–1310.e20 (2020).

doi:10.1016/j.cell.2020.08.012 Medline

18. J. Zahradník, S. Marciano, M. Shemesh, E. Zoler, D. Harari, J. Chiaravalli, B. Meyer, Y. Rudich, C. Li, I. Marton, O. Dym, N. Elad, M. G. Lewis, H. Andersen, M. Gagne, R. A. Seder, D. C. Douek, G. Schreiber, SARS-CoV-2 variant prediction and antiviral drug design are enabled by RBD in vitro evolution. *Nat. Microbiol.* **6**, 1188–1198 (2021). doi:10.1038/s41564-021-00954-4 Medline
19. E. Cameroni, J. E. Bowen, L. E. Rosen, C. Saliba, S. K. Zepeda, K. Culap, D. Pinto, L. A. VanBlargan, A. De Marco, J. di Iulio, F. Zatta, H. Kaiser, J. Noack, N. Farhat, N. Czudnochowski, C. Havenar-Daughton, K. R. Sprouse, J. R. Dillen, A. E. Powell, A. Chen, C. Maher, L. Yin, D. Sun, L. Soriaga, J. Bassi, C. Silacci-Fregni, C. Gustafsson, N. M. Franko, J. Logue, N. T. Iqbal, I. Mazzitelli, J. Geffner, R. Grifantini, H. Chu, A. Gori, A. Riva, O. Giannini, A. Ceschi, P. Ferrari, P. E. Cippà, A. Franzetti-Pellanda, C. Garzoni, P. J. Halfmann, Y. Kawaoka, C. Hebner, L. A. Purcell, L. Piccoli, M. S. Pizzuto, A. C. Walls, M. S. Diamond, A. Telenti, H. W. Virgin, A. Lanzavecchia, G. Snell, D. Veesler, D. Corti, Broadly neutralizing antibodies overcome SARS-CoV-2 Omicron antigenic shift. *Nature* 10.1038/s41586-021-04386-2 (2021). doi:10.1038/s41586-021-04386-2 Medline
20. J. W. Saville, D. Mannar, X. Zhu, S. S. Srivastava, A. M. Berezuk, J.-P. Demers, S. Zhou, K. S. Tuttle, I. Sekirov, A. Kim, W. Li, D. S. Dimitrov, S. Subramaniam, Structural and biochemical rationale for enhanced spike protein fitness in Delta and Kappa SARS-CoV-2 variants. bioRxiv 2021.2009.2002.458774 [Preprint] (2021); https://doi.org/10.1101/2021.09.02.458774.
21. F. Krammer, SARS-CoV-2 vaccines in development. *Nature* **586**, 516–527 (2020). doi:10.1038/s41586-020-2798-3 Medline
22. W. Li, C. Chen, A. Drelich, D. R. Martinez, L. E. Gralinski, Z. Sun, A. Schäfer, S. S. Kulkarni, X. Liu, S. R. Leist, D. V. Zhelev, L. Zhang, Y.-J. Kim, E. C. Peterson, A. Conard, J. W. Mellors, C. K. Tseng, D. Falzarano, R. S. Baric, D. S. Dimitrov, Rapid identification of a human antibody with high prophylactic and therapeutic efficacy in three animal models of SARS-CoV-2 infection. *Proc. Natl. Acad. Sci. U.S.A.* **117**, 29832–29838 (2020). doi:10.1073/pnas.2010197117 Medline
23. W. Li, A. Schäfer, S. S. Kulkarni, X. Liu, D. R. Martinez, C. Chen, Z. Sun, S. R. Leist, A. Drelich, L. Zhang, M. L. Ura, A. Berezuk, S. Chittori, K. Leopold, D. Mannar, S. S. Srivastava, X. Zhu, E. C. Peterson, C.-T. Tseng, J. W. Mellors, D. Falzarano, S. Subramaniam, R. S. Baric, D. S. Dimitrov, High potency of a bivalent human V_H domain in SARS-CoV-2 animal models. *Cell* **183**, 429–441.e16 (2020). doi:10.1016/j.cell.2020.09.007 Medline
24. M. A. Tortorici, M. Beltramello, F. A. Lempp, D. Pinto, H. V. Dang, L. E. Rosen, M. McCallum, J. Bowen, A. Minola, S. Jaconi, F. Zatta, A. De Marco, B. Guarino, S. Bianchi, E. J. Lauron, H. Tucker, J. Zhou, A. Peter, C. Havenar-Daughton, J. A. Wojcechowskyj, J. B. Case, R. E. Chen, H. Kaiser, M. Montiel-Ruiz, M. Meury, N. Czudnochowski, R. Spreafico, J. Dillen, C. Ng, N. Sprugasci, K. Culap, F. Benigni, R. Abdelnabi, S. C. Foo, M. A. Schmid, E. Cameroni, A. Riva, A. Gabrieli, M. Galli, M. S. Pizzuto, J. Neyts, M. S. Diamond, H. W. Virgin, G. Snell, D. Corti, K. Fink, D. Veesler, Ultrapotent human antibodies protect against SARS-CoV-2 challenge via multiple mechanisms. *Science* **370**, 950–957 (2020). doi:10.1126/science.abe3354 Medline
25. D. Pinto, Y.-J. Park, M. Beltramello, A. C. Walls, M. A. Tortorici, S. Bianchi, S. Jaconi, K. Culap, F. Zatta, A. De Marco, A. Peter, B. Guarino, R. Spreafico, E. Cameroni, J. B. Case, R. E. Chen, C. Havenar-Daughton, G. Snell, A. Telenti, H. W. Virgin, A. Lanzavecchia, M. S. Diamond, K. Fink, D. Veesler, D. Corti, Cross-neutralization of SARS-CoV-2 by a human monoclonal SARS-CoV antibody. *Nature* **583**, 290–295 (2020). doi:10.1038/s41586-020-2349-y Medline
26. L. Liu, P. Wang, M. S. Nair, J. Yu, M. Rapp, Q. Wang, Y. Luo, J. F.-W. Chan, V. Sahi, A. Figueroa, X. V. Guo, G. Cerutti, J. Bimela, J. Gorman, T. Zhou, Z. Chen, K.-Y. Yuen, P. D. Kwong, J. G. Sodroski, M. T. Yin, Z. Sheng, Y. Huang, L. Shapiro, D. D. Ho, Potent neutralizing antibodies against multiple epitopes on SARS-CoV-2 spike. *Nature* **584**, 450–456 (2020). doi:10.1038/s41586-020-2571-7 Medline
27. X. Chi, R. Yan, J. Zhang, G. Zhang, Y. Zhang, M. Hao, Z. Zhang, P. Fan, Y. Dong, Y. Yang, Z. Chen, Y. Guo, J. Zhang, Y. Li, X. Song, Y. Chen, L. Xia, L. Fu, L. Hou, J. Xu, C. Yu, J. Li, Q. Zhou, W. Chen, A neutralizing human antibody binds to the N-terminal domain of the Spike protein of SARS-CoV-2. *Science* **369**, 650–655 (2020). doi:10.1126/science.abc6952 Medline
28. D. Mannar, J. W. Saville, Z. Sun, X. Zhu, M. M. Marti, S. S. Srivastava, A. M. Berezuk, S. Zhou, K. S. Tuttle, M. D. Sobolewski, A. Kim, B. R. Treat, P. M. Da Silva Castanha, J. L. Jacobs, S. M. Barratt-Boyes, J. W. Mellors, D. S. Dimitrov, W. Li, S. Subramaniam, Emerging SARS-CoV-2 variants of concern: Spike protein mutational analysis and epitope for broad neutralization. bioRxiv 2021.12.17.473178 [Preprint] (2021); https://doi.org/10.1101/2021.12.17.473178.
29. L. VanBlargan, J. M. Errico, P. J. Halfmann, S. J. Zost, J. E. Crowe Jr., L. A. Purcell, Y. Kawaoka, D. Corti, D. H. Fremont, M. S. Diamond, An infectious SARS-CoV-2 B.1.1.529 Omicron virus escapes neutralization by several therapeutic monoclonal antibodies. bioRxiv 2021.2012.2015.472828 [Preprint] (2021); https://doi.org/10.1101/2021.12.15.472828.
30. A. Cathcart, C. Havenar-Daughton, F. A. Lempp, D. Ma, M. A. Schmid, M. L. Agostini, B. Guarino, J. Diiulio, L. E. Rosen, H. Tucker, J. Dillen, S. Subramanian, B. Sloan, S. Bianchi, D. Pinto, C. Saliba, K. Culap, J. A. Wojcechowskyj, J. Noack, J. Zhou, H. Kaiser, A. Chase, M. Montiel-Ruiz, E. Dellota Jr., A. Park, R. Spreafico, A. Sahakyan, E. J. Lauron, N. Czudnochowski, E. Cameroni, S. Ledoux, A. Werts, C. Colas, L. Soriaga, A. Telenti, L. A. Purcell, S. Hwang, G. Snell, H. W. Virgin, D. Corti, C. M. Hebner, The dual function monoclonal antibodies VIR-7831 and VIR-7832 demonstrate potent in vitro and in vivo activity against SARS-CoV-2. bioRxiv 2021.03.09.434607 [Preprint] (2021); https://doi.org/10.1101/2021.03.09.434607.
31. M. McCallum, N. Czudnochowski, L. E. Rosen, S. K. Zepeda, J. E. Bowen, J. R. Dillen, A. E. Powell, T. I. Croll, J. Nix, H. W. Virgin, D. Corti, G. Snell, D. Veesler, Structural basis of SARS-CoV-2 Omicron immune evasion and receptor engagement. bioRxiv 2021.2012.2028.474380 [Preprint] (2021); https://doi.org/10.1101/2021.12.28.474380.
32. D. Planas, N. Saunders, P. Maes, F. Guivel-Benhassine, C. Planchais, J. Buchrieser, W. H. Bolland, F. Porrot, I. Staropoli, F. Lemoine, H. Péré, D. Veyer, J. Puech, J. Rodary, G. Baele, S. Dellicour, J. Raymenants, S. Gorissen, C. Geenen, B. Vanmechelen, T. Wawina-Bokalanga, J. Martí-Carreras, L. Cuypers, A. Sève, L. Hocqueloux, T. Prazuck, F. Rey, E. Simon-Loriere, T. Bruel, H. Mouquet, E. André, O. Schwartz, Considerable escape of SARS-CoV-2 Omicron to antibody neutralization. *Nature* 10.1038/s41586-021-04389-z (2021). doi:10.1038/s41586-021-04389-z Medline
33. A. Rössler, L. Riepler, D. Bante, D. v. Laer, J. Kimpel, SARS-CoV-2 B.1.1.529 variant (Omicron) evades neutralization by sera from vaccinated and convalescent individuals. medRxiv 2021.2012.2008.21267491 [Preprint] (2021); https://doi.org/10.1101/2021.12.08.21267491.
34. L. Liu, S. Iketani, Y. Guo, J. F.-W. Chan, M. Wang, L. Liu, Y. Luo, H. Chu, Y. Huang, M. S. Nair, J. Yu, K. K.-H. Chik, T. T.-T. Yuen, C. Yoon, K. K.-W. To, H. Chen, M. T. Yin, M. E. Sobieszczyk, Y. Huang, H. H. Wang, Z. Sheng, K.-Y. Yuen, D. D. Ho, Striking antibody evasion manifested by the Omicron variant of SARS-CoV-2. *Nature* 10.1038/s41586-021-04388-0 (2021). doi:10.1038/s41586-021-04388-0 Medline
35. A. Punjani, J. L. Rubinstein, D. J. Fleet, M. A. Brubaker, cryoSPARC: Algorithms for rapid unsupervised cryo-EM structure determination. *Nat. Methods* **14**, 290–296 (2017). doi:10.1038/nmeth.4169 Medline
36. E. F. Pettersen, T. D. Goddard, C. C. Huang, G. S. Couch, D. M. Greenblatt, E. C. Meng, T. E. Ferrin, UCSF Chimera—A visualization system for exploratory research and analysis. *J. Comput. Chem.* **25**, 1605–1612 (2004). doi:10.1002/jcc.20084 Medline
37. P. Emsley, B. Lohkamp, W. G. Scott, K. Cowtan, Features and development of Coot. *Acta Crystallogr. D Biol. Crystallogr.* **66**, 486–501 (2010). doi:10.1107/S0907444910007493 Medline
38. D. Liebschner, P. V. Afonine, M. L. Baker, G. Bunkóczi, V. B. Chen, T. I. Croll, B. Hintze, L.-W. Hung, S. Jain, A. J. McCoy, N. W. Moriarty, R. D. Oeffner, B. K. Poon, M. G. Prisant, R. J. Read, J. S. Richardson, D. C. Richardson, M. D. Sammito, O. V. Sobolev, D. H. Stockwell, T. C. Terwilliger, A. G. Urzhumtsev, L. L. Videau, C. J. Williams, P. D. Adams, Macromolecular structure determination using x-rays, neutrons and electrons: Recent developments in Phenix. *Acta Crystallogr. D Struct. Biol.* **75**, 861–877 (2019). doi:10.1107/S2059798319011471 Medline
39. T. D. Goddard, C. C. Huang, E. C. Meng, E. F. Pettersen, G. S. Couch, J. H. Morris, T. E. Ferrin, UCSF ChimeraX: Meeting modern challenges in visualization and analysis. *Protein Sci.* **27**, 14–25 (2018). doi:10.1002/pro.3235 Medline

ACKNOWLEDGMENTS

We thank Dr. Karoline Leopold (UBC) and Dr. Charles Leung (Gandeeva Therapeutics Inc.) for assistance with the surface plasmon resonance experiments and for helpful discussions. **Funding:** This work was supported by awards to S.S. from a Canada Excellence Research Chair Award, the VGH Foundation, Genome BC, Canada, and from the Tai Hung Fai Charitable Foundation. D.M. is supported by a CIHR Frederick Banting and Charles Best Canada Graduate Scholarship Master's Award (CGS-M). J.W.S is supported by a CIHR Frederick Banting and Charles Best Canada Graduate Scholarships Doctoral Award (CGS D) and a UBC President's Academic Excellence Initiative PhD Award. **Author contributions:** This work was the result of a concerted team effort from all individuals listed as authors. J.W.S. and D.M., carried out expression and purification of the Omicron spike protein and antibodies. D.M. performed the SPR binding analyses. D.M. and J.W.S. performed the pseudovirus neutralization experiments. I.S. and C.M. provided the vaccine-induced patient-derived sera samples and aided the interpretation of the patient data. A.M.B., S.S.S. and K.S.T. carried out experimental aspects of electron microscopy including specimen preparation and data collection. X.Z. carried out computational aspects of image processing and structure determination. X.Z., S.S.S., D.M., J.W.S., and S.S. interpreted and analyzed the cryo-EM structures, binding analyses and patient neutralization data and composed the manuscript with input from the rest of the authors. S.S. provided overall supervision for the project. **Competing interests:** All authors except for S.S. declare no competing interests. S.S. is the Founder and CEO of Gandeeva Therapeutics Inc. **Data and materials availability:** All newly created materials described in this manuscript will be available from the corresponding author upon reasonable request. Cryo-EM reconstructions and atomic models generated during this study are available at the PDB and EMBD databases under the following accession codes: Unbound Omicron spike protein trimer (PDB ID 7T9J, EMD- EMDB 25759), global ACE2-bound Omicron spike protein trimer (PDB ID 7T9K, EMD-25760), focus-refinement of the ACE2-RBD interface for the ACE2-bound Omicron spike protein trimer (PDB ID 7T9L, EMD-25761). This work is licensed under a Creative Commons Attribution 4.0 International (CC BY 4.0) license, which permits unrestricted use, distribution, and reproduction in any medium, provided the original work is properly cited. To view a copy of this license, visit https://creativecommons.org/licenses/by/4.0/. This license does not apply to figures/photos/artwork or other content included in the article that is credited to a third party; obtain authorization from the rights holder before using such material.

SUPPLEMENTARY MATERIALS

science.org/doi/10.1126/science.abn7760
Materials and Methods
Figs. S1 to S9
Tables S1 to S3
References (*35*–*39*)
MDAR Reproducibility Checklist

19 December 2021; accepted 18 January 2022
Published online 20 January 2022
10.1126/science.abn7760

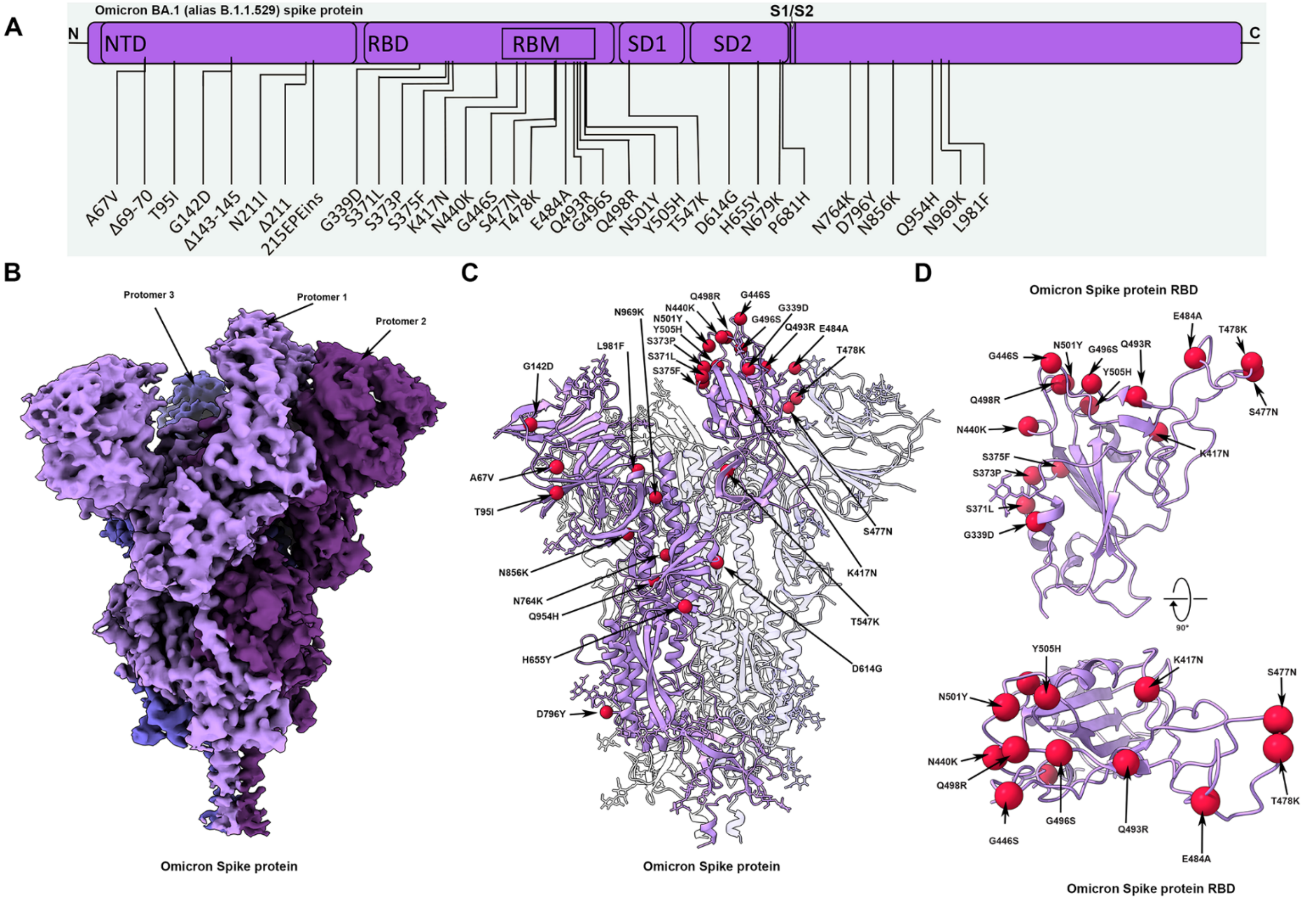

Fig. 1. Cryo-EM structure of the Omicron spike protein. (A) A schematic diagram illustrating the domain arrangement of the spike protein. Mutations present in the Omicron variant spike protein are labeled. (B) Cryo-EM map of the Omicron spike protein at 2.79 Å resolution. Protomers are colored in different shades of purple. (C) Cryo-EM structure of Omicron spike protein indicating the locations of modeled mutations on one protomer. (D) The Omicron spike receptor-binding domain (RBD) shown in two orthogonal orientations with Cα positions of the mutated residues shown as red spheres.

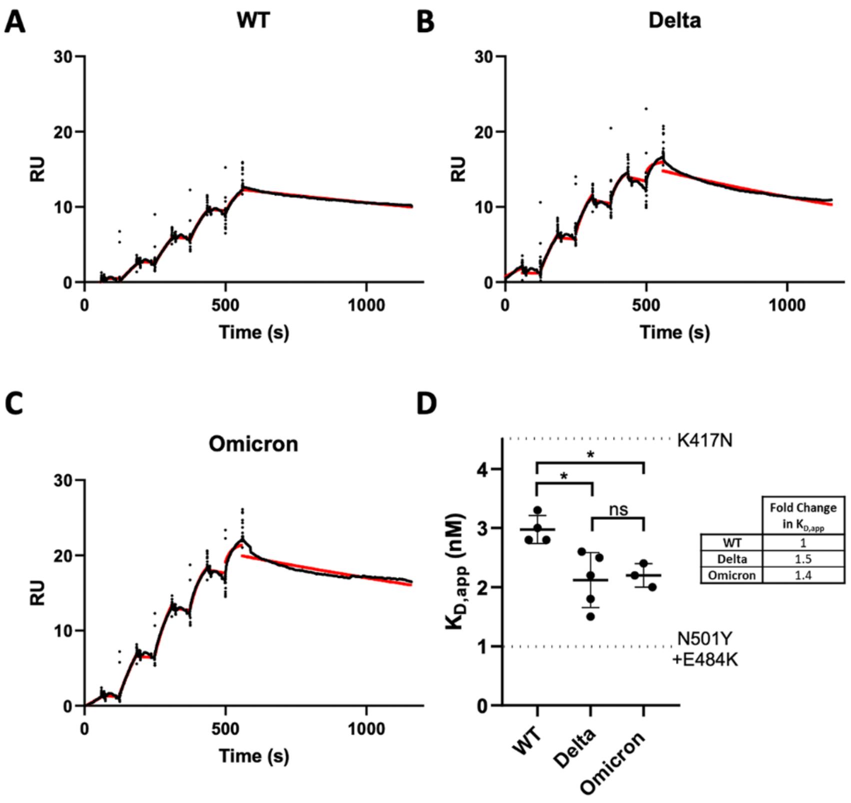

Fig. 2. SPR analysis of the wild-type, Delta, and Omicron spike protein affinities for human ACE2. (**A** to **C**) Representative traces of single-cycle kinetic analyses of S protein-ACE2 binding. The raw data (black) is fit (red) to a model using a 1:1 binding stoichiometry from which apparent dissociation constants were derived. The curves were obtained by injecting 6.25, 31.25, 62.5, 125, 250 nM of each spike protein in successive cycles. (RU: Response units). (**D**) Quantitation of apparent dissociation constants ($K_{D,app}$) for the wild-type, Delta, and Omicron S protein-ACE2 interaction. The standard deviation obtained from at least three technical replicates is shown. Horizontal dotted lines are plotted for mutants carrying only K417N (top) or N501Y + E484K (bottom) mutations to demonstrate the range of this assay (see fig. S2 for binding data). A Tukey's multiple comparisons test was performed on the wild-type, Delta, and Omicron binding affinities (*$P \leq 0.05$, ns = not significant). A table highlighting the fold changes in $K_{D,app}$ for the Delta and Omicron S protein-ACE2 interactions relative to WT is shown.

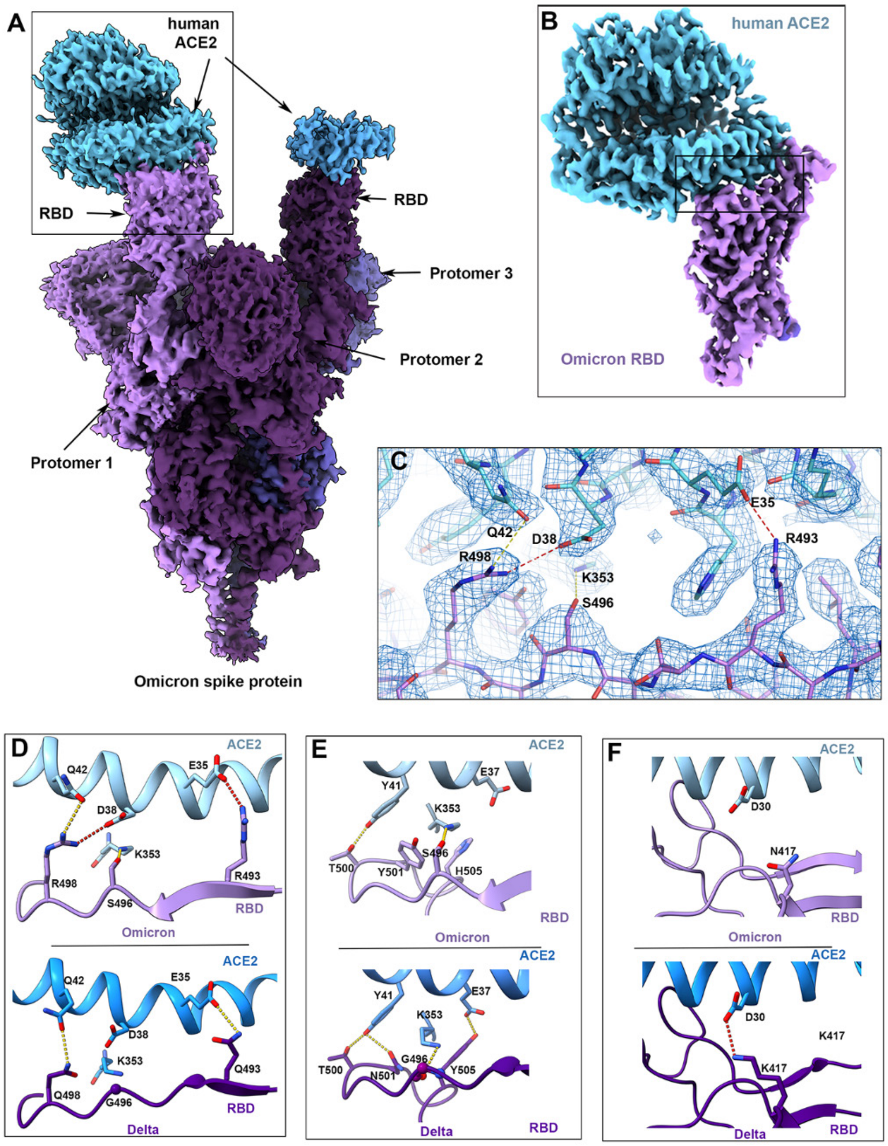

Fig. 3. Cryo-EM structure of the Omicron spike protein-ACE2 complex. (A) Cryo-EM map of the Omicron spike protein in complex with human ACE2 at 2.45 Å resolution after global refinement. The three protomers are colored in different shades of purple and the density for bound ACE2 is colored in blue. (B) Cryo-EM map of the Omicron spike RBD in complex with ACE2 at 2.66 Å resolution after focused refinement. The inset box indicates the region highlighted in (C). (C) Cryo-EM density mesh at the Omicron spike RBD-ACE2 interface, with fitted atomic model. Yellow and red dashed lines represent new hydrogen bonds and ionic interactions, respectively. (D to F) Comparison of the RBD-ACE2 interface between the Omicron (upper) and Delta (lower) variants. Compared to the Delta variant, new interactions are formed as a result of the mutations Q493R, G496S, and Q498R (D), and local structural changes due to the N501Y and Y505H mutations present (E) in the Omicron variant. The salt bridge between Delta RBD K417 and ACE2 D30 that is present in the Delta variant spike protein but lost in the Omicron variant is highlighted in (F). Yellow and red dashed lines represent hydrogen bonds and ionic interactions, respectively.

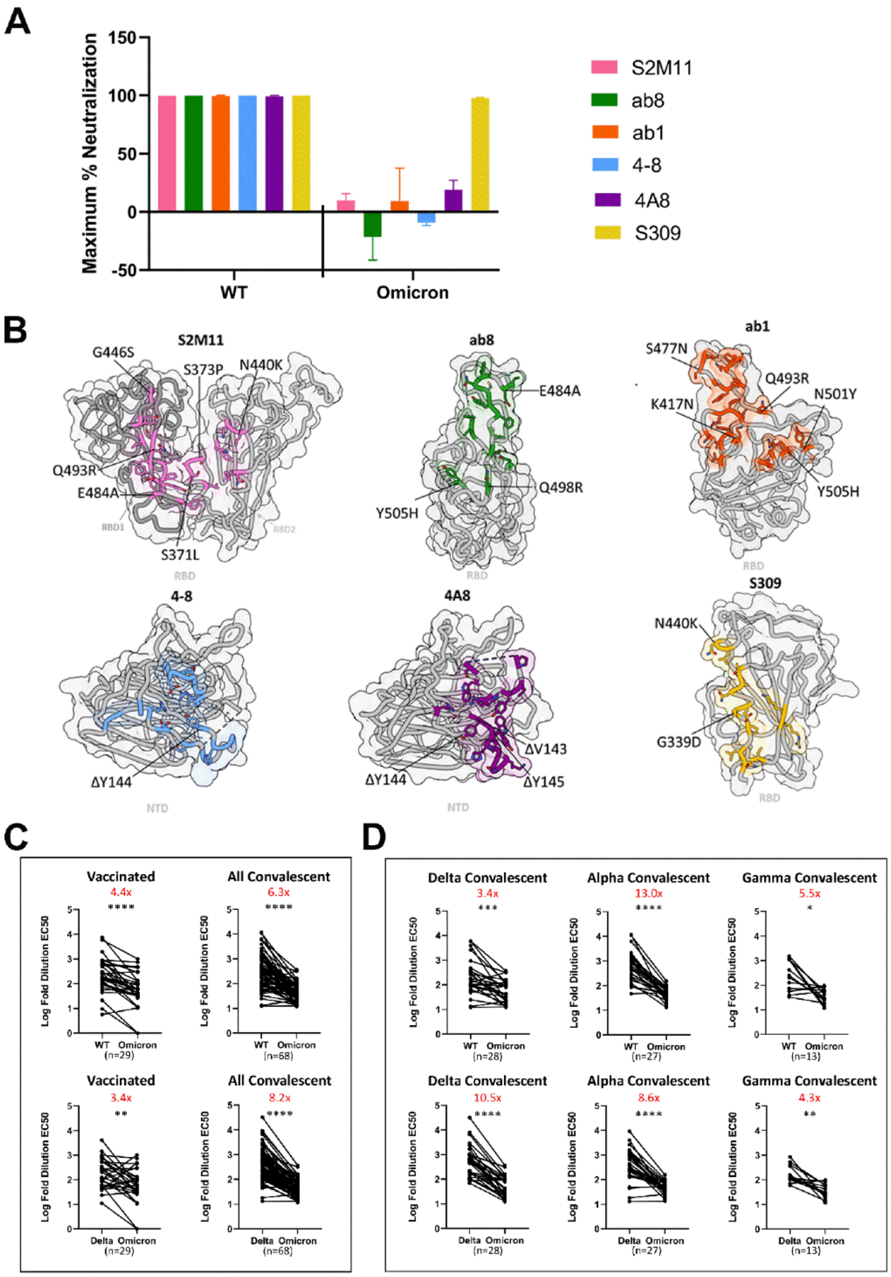

Fig. 4. Monoclonal antibodies and vaccinated and convalescent patient-derived sera exhibit decreased Omicron neutralization potency. (**A**) Maximum neutralization achieved by the indicated monoclonal antibodies against wild-type and Omicron pseudoviruses (n = 3 technical replicates). Error bars denote standard deviation of the mean. (**B**) Antibody binding footprints for monoclonal antibodies tested in this study. Omicron spike protein mutations falling within each antibody footprint are labeled. (**C**) Log-fold EC50 dilutions for vaccinated and convalescent patient sera for either wild-type (WT) versus Omicron variant pseudoviruses (top) or Delta and Omicron variant pseudoviruses (bottom). (**D**) As in (C) with a breakdown of the convalescent patients into previous infection with Delta, Alpha, and Gamma variants of concern. A pairwise statistical significance test was performed using the Wilcoxon matched pairs test (*P≤0.05, **P≤0.01, ***P≤0.001, ****P≤0.0001). The fold-change in the geometric mean between the two groups is shown in red text at the top of each plot.
