# Supplementary material for: SARS-CoV-2 Omicron variant: Antibody evasion and cryo-EM structure of spike protein–ACE2 complex
Source: Science. 2022 Jan 20;375(6582):760–4. doi: 10.1126/science.abn7760 (PMC9799367; doi:10.1126/science.abn7760)
Supplement: Supplementary file 2 — Materials and Methods Figs. S1 to S9 Tables S1 to S3 References (35–39) [file science.abn7760_sm.pdf]

Science
AAAS

# Supplementary Materials for

## SARS-CoV-2 Omicron variant: Antibody evasion and cryo-EM structure of spike protein–ACE2 complex

Dhiraj Mannar *et al*.

Corresponding author: Sriram Subramaniam, sriram.subramaniam@ubc.ca

*Science* **375**, 760 (2022)
DOI: 10.1126/science.abn7760

**The PDF file includes:**

Materials and Methods
Figs. S1 to S9
Tables S1 to S3
References

**Other Supplementary Material for this manuscript includes the following:**

MDAR Reproducibility Checklist

## Materials and Methods

### Ethics Statement

Patient derived sera samples were collected according to the CARE COVID Study (http://www.bccdc.ca/health-professionals/clinical-resources/covid-19-care/covid-19-serology-care-covid-study) with ethics approval from the UBC Clinical Research Ethics Board (H21-01828).

### Expression and Purification of Omicron Recombinant Spike Protein Constructs

For the ACE2 binding and structural studies, we utilized HexaPro constructs, which differ from native S proteins by the introduction of six stabilizing proline substitutions (F817P, A892P, A899P, A942P, K968P, V969P) along with the replacement of the transmembrane domain with a trimerization motif. These protein modifications result in higher yields during mammalian expression and increased thermostability. The production of the SARS-CoV-2 wild-type (D614G), K417N, N501Y+E484K and Delta HexaPro S proteins were described previously *(12,19).*The SARS-CoV-2 Omicron HexaPro S protein gene was synthesized and inserted into pcDNA3.1 (GeneArt Gene Synthesis, Thermo Fisher Scientific). Expi293F cells (Thermo Fisher, Cat# A14527) were grown in suspension culture using Expi293 Expression Medium (Thermo Fisher, Cat# A1435102) at 37°C, 8% $CO_2$. Cells were transiently transfected at a density of 3 x 10^6 cells/mL using linear polyethylenimine (Polysciences Cat# 23966-1). The media was supplemented 24 hours after transfection with 2.2 mM valproic acid, and expression was carried out for 3 days at 37°C, 8% $CO_2$. The supernatant was harvested by centrifugation and filtered through a 0.22-µM filter prior to loading onto a 5 mL HisTrap excel column (Cytiva). The column was washed for 20 CVs with wash buffer (20 mM Tris pH 8.0, 500 mM NaCl), 5 CVs of wash buffer supplemented with 20 mM imidazole, and the protein eluted with elution buffer (20 mM Tris pH 8.0, 500 mM NaCl, 500 mM imidazole). Elution fractions containing the protein were pooled and concentrated (Amicon Ultra 100 kDa cut off, Millipore Sigma) for gel filtration. Gel filtration was conducted using a Superose 6 10/300 GL column (Cytiva) pre-equilibrated with GF buffer (20 mM Tris pH 8.0, 150 mM NaCl). Peak fractions corresponding to soluble protein were pooled and concentrated to 4.5–6.5 mg/mL (Amicon Ultra 100 kDa cut off, Millipore Sigma). Protein samples were flash-frozen in liquid nitrogen and stored at -80°C.

### Antibody Production

VH-FC ab8, IgG ab1, Fab S309, Fab S2M11, Fab 4-8, and Fab 4A8 were produced as previously described *(12,20)*.

### Surface Plasmon Resonance

SPR experiments were performed on the Biacore T200 instrument. Recombinant human ACE2 attached to a mouse Fc fragment (ACE2-mFc) (SinoBiological, cat# 10108-H05H, Accession # NP_068576.1) was immobilized using the series S protein A chip. Increasing concentrations (6.25nM, 31.25nM, 62.5nM, 125nM, 250nM) of various spike protein trimers were flowed over the surface for single cycle kinetic experiments. The surface was regenerated in 10mM glycine pH 1. The experiments were performed at 25 degrees Celsius, using 10mM HEPES, 150mM NaCl, 3mM EDTA and 0.05% v/v Surfactant P20 as running buffer. Reference-subtracted curves were fitted to a 1:1 binding model using Biacore evaluation software.

**Pseudovirus Neutralization Assay**

SARS-CoV-2 S protein Omicron genes were synthesized and inserted into pcDNA3.1 (GeneArt Gene Synthesis, Thermo Fisher Scientific). Pseudotyped retroviral particles were produced in HEK293T cells (ATCC, cat#CRL-3216) as described previously *(12)*. Briefly, a lentiviral packaging system was utilized in combination with plasmids encoding the full-length SARS-CoV-2 wild-type (D614G), Delta, and Omicron spikes, along with a transfer plasmid encoding luciferase and GFP as a dual reporter gene. Pseudoviruses were harvested 60 h after transfection and filtered with a 0.45 µm PES filter. For neutralization assays, HEK293T-ACE2-TMPRSS2 cells (BEI Resources cat# NR-55293) were seeded in 384-well plates at 20 000 cells. The next day, pseudovirus preparations normalized for viral capsid p24 levels (Lenti-X™ GoStix™ Plus) were incubated with dilutions of the indicated antibodies, sera, or media alone for 1 h at 37°C prior to addition to cells and incubation for 48 h. Cells were then lysed and luciferase activity assessed using the ONE-Glo™ EX Luciferase Assay System (Promega) according to the manufacturer's specifications. Detection of relative luciferase units was carried out using a Varioskan Lux plate reader (Thermo Fisher).

**Electron Microscopy Sample Preparation and Data Collection**

For cryo-EM, 2.25 mg/mL S protein and S protein – human ACE2 (residues 1-615) complex (1:2.3 S protein trimer:ACE2 molar ratio) samples were vitrified using a Vitrobot Mark IV (Thermo Fisher Scientific) plunge freezing device. Quantifoil R1.2/1.3 Cu mesh 200 holey carbon grids were first glow discharged for 20 seconds using a Pelco easiGlow glow discharge unit (Ted Pella) and then 1.8 µL of protein suspension was applied to the surface of the grid at a temperature of 10°C and a humidity level of >98%. Grids were then blotted (12 sec, blot force -10) and plunge frozen into liquid ethane. S protein-ACE2 complex grids were imaged using a 300 kV Titan Krios G4 transmission electron microscope (Thermo Fisher Scientific) equipped with a Falcon4 direct electron detector in electron event registration (EER) mode. Movies were collected at 155,000x magnification (calibrated pixel size of 0.5 Å per physical pixel) over a defocus range of -0.5 µm to -2 µm with a total dose of 40 $e^-/Å^2$ using EPU automated acquisition software. Grids containing the Omicron S protein alone were imaged using a 200 kV Glacios transmission electron microscope (Thermo Fisher Scientific) equipped with a Falcon4 camera operated in EER mode. Micrographs were collected using EPU at 190,000x magnification (physical pixel size 0.7 Å) over a defocus range of -0.5 µm to -2 µm with a total accumulated dose of 40 $e^-/Å^2$.

**Image Processing**

The detailed data processing workflow is summarized in Supplementary Figures S1,S3. All data processing was done in cryoSPARC v.3.3.1 *(35)*. Motion correction in patch mode (EER upsampling factor 1, EER number of fractions 40), CTF estimation in patch mode, blob particle picking, and particle extraction (box size 400 Å) were performed on-the-fly in cryoSPARC. Then particles were subjected to multiple rounds of 3D heterogeneous classification. The final 3D refinement was performed with per particle CTF estimation and aberration correction. For the complexes of Omicron spike protein ectodomain and human ACE2, local refinement was performed with a soft mask covering a single RBD and its bound ACE2.

**Model Building and Refinement**

For models of Omicron spike protein ectodomain alone, the SARS-CoV-2 HexaPro S trimer with N501Y mutation (PDB code 7MJG) was fitted into the map using UCSF Chimera v.1.15 *(36)*.

Then, mutation and manual adjustment were carried out with COOT v.0.9.3 *(37)*, followed by iterative rounds of real-space refinement in COOT and Phenix v.1.19 *(38)*. Glycans were added at N-linked glycosylation sites in COOT. For models of Omicron spike-ACE2 complex, the RBD-ACE2 subcomplex was built using published coordinates (PDB code 7MJN) as the initial model, followed by refinement against local refinement maps. The obtained model was then docked back into global refinement maps together with the other individual domains of the spike protein. Model validation was performed using MolProbity *(38)*. Figures were prepared using UCSF Chimera, UCSF ChimeraX v.1.1.1 *(39)*, and PyMOL (v.2.2 Schrodinger, LLC).

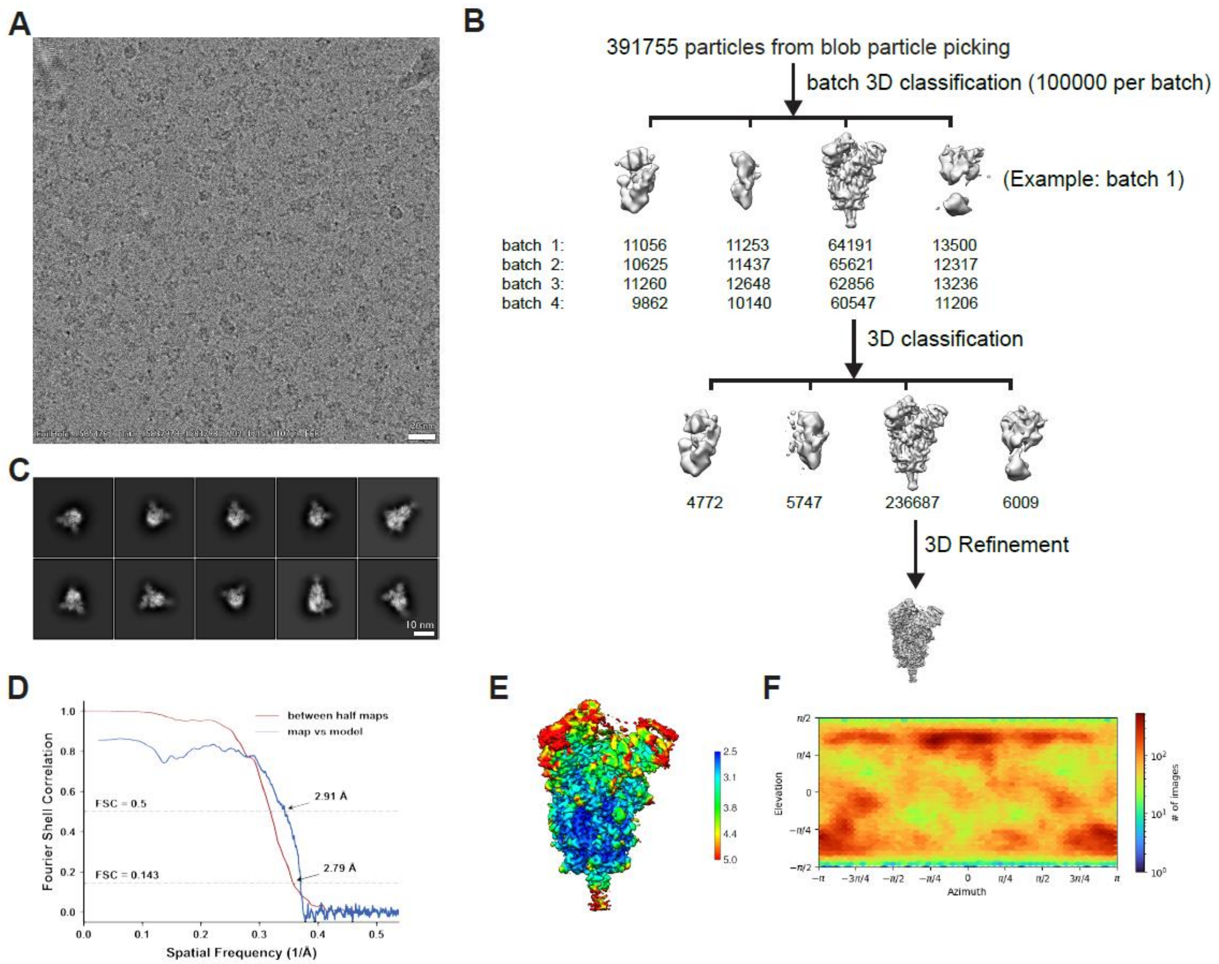


**Fig. S1. Cryo-EM data processing and validation for the Omicron spike protein ectodomain.** (A) Representative cryo-EM micrograph. (B) Workflow of cryo-EM image processing. (C) Representative 2D classes. (D) FSC curves. (E) Local resolution. (F) Viewing direction distribution plot.

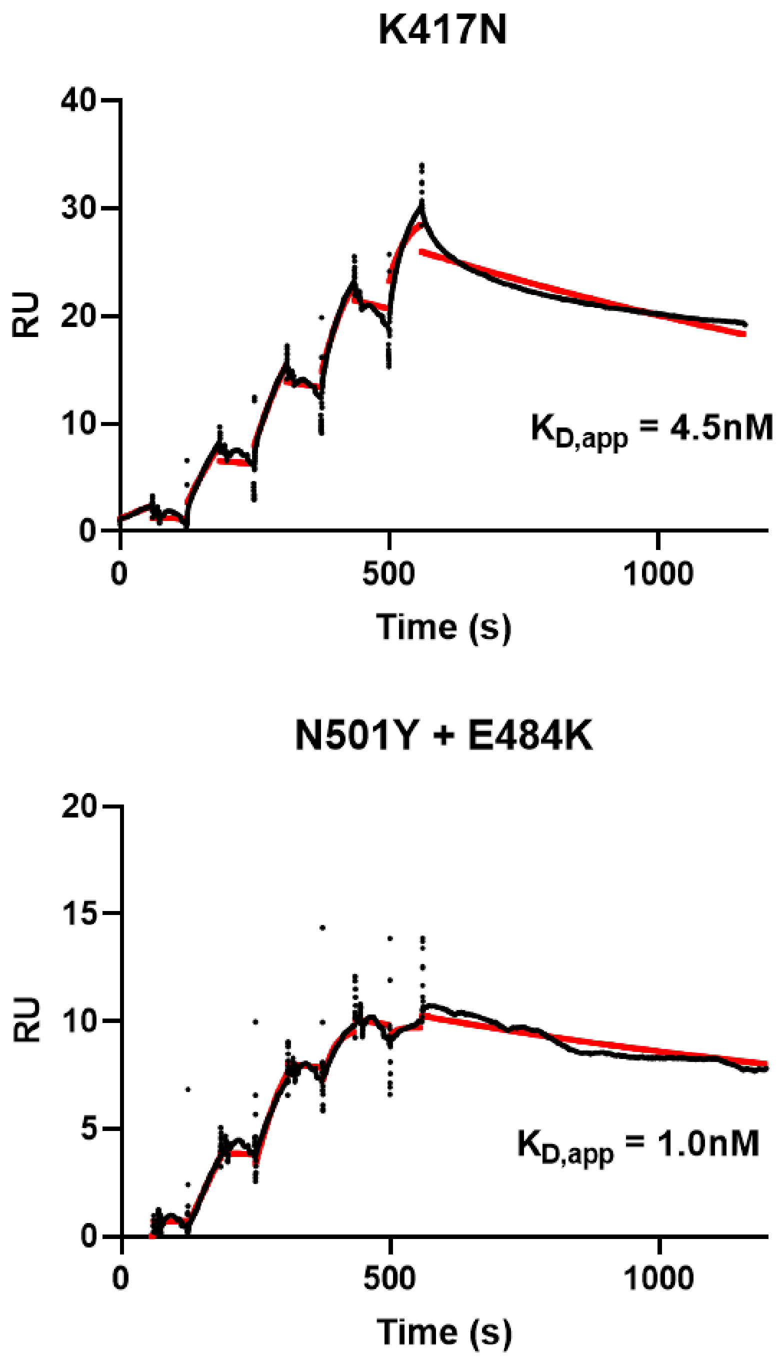


**Fig. S2. SPR Analysis of the K417N and N501Y+E484K spike protein affinities for ACE2.** Red curves show the fitting to the raw data (shown in black) using a 1:1 binding model. 6.25, 31.25, 62.5, 125, 250 nM of each spike protein was injected in successive cycles. (RU: Response units). Apparent dissociation constants ($K_{D,app}$) are reported for each experiment.

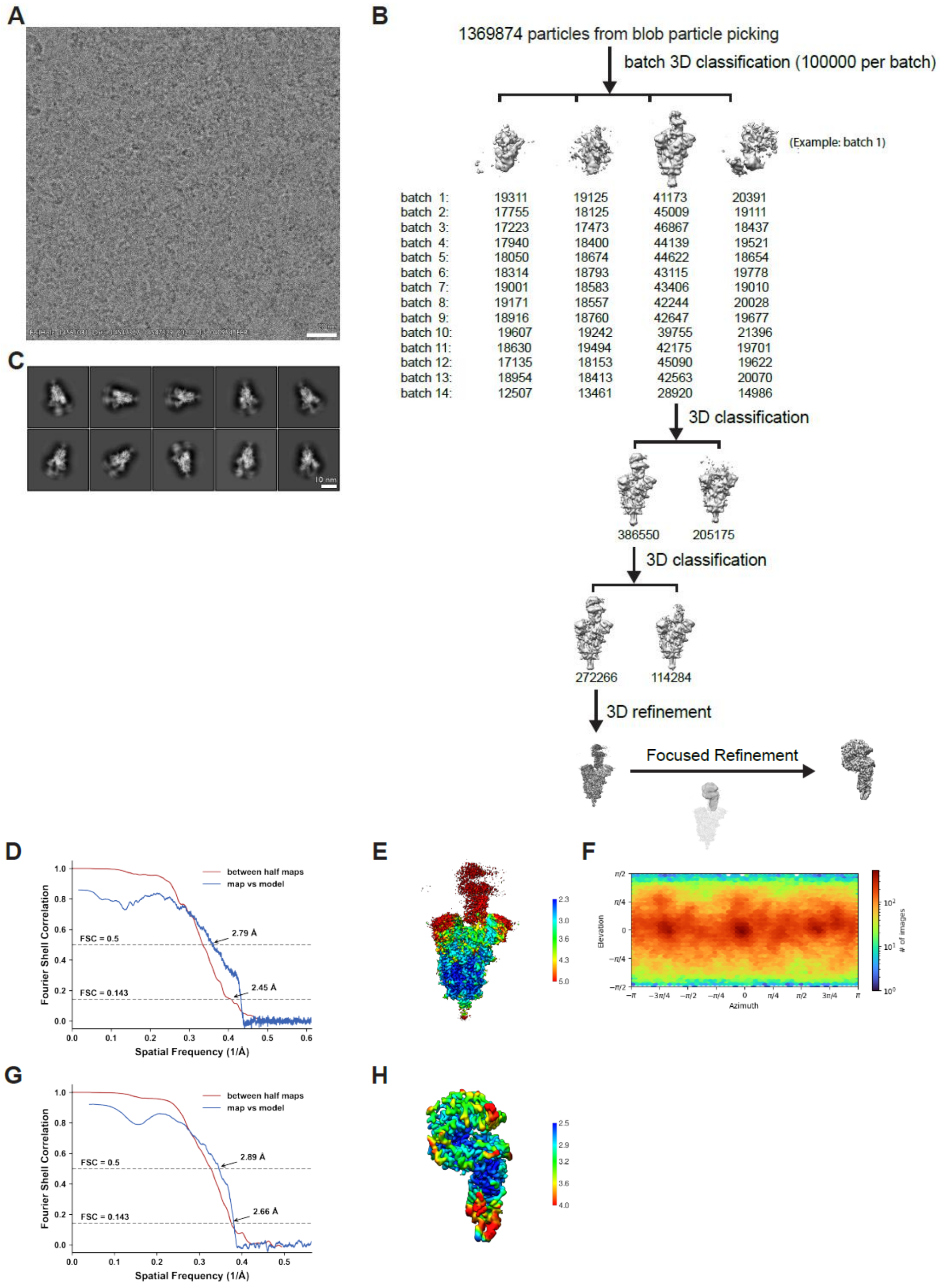


**Fig. S3. Cryo-EM data processing and validation for complex of Omicron spike protein ectodomain and human ACE2.** (A) Representative cryo-EM micrograph. (B) Workflow of cryo-EM image processing. (C) Representative 2D classes. (D-F) FSC curves (D), local resolution (E) and viewing direction distribution plot (F) of global refinement. (G-H) FSC curves (G) and local resolution (H) of focused refinement.

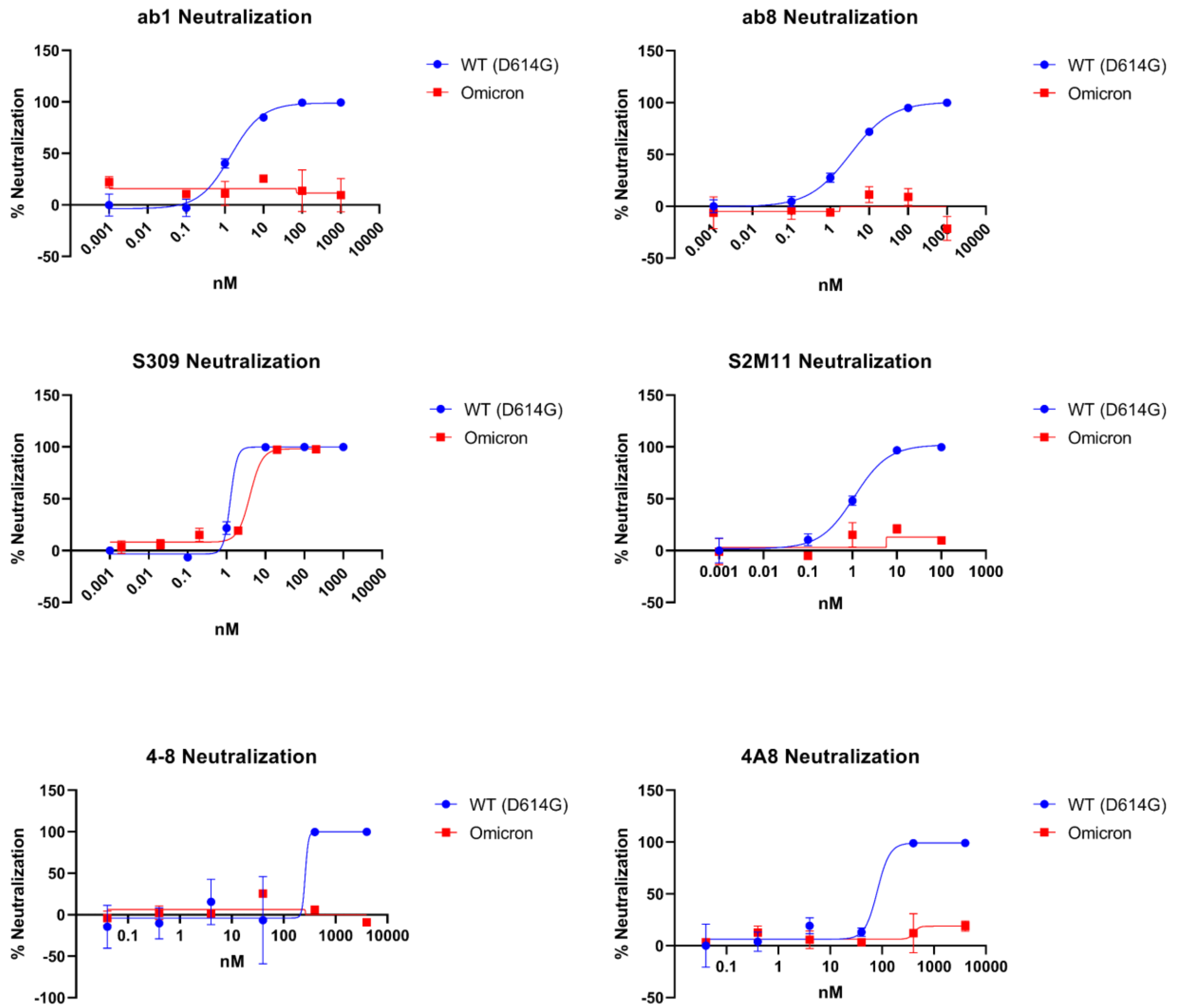


**Fig. S4. Monoclonal antibody neutralization of Omicron S protein pseudotyped viruses and comparison to previously determined *(20)* wild-type S protein pseudotyped virus neutralization curves.** Points denote the mean of (n = 3) replicates, error bars denote the standard error of the mean.

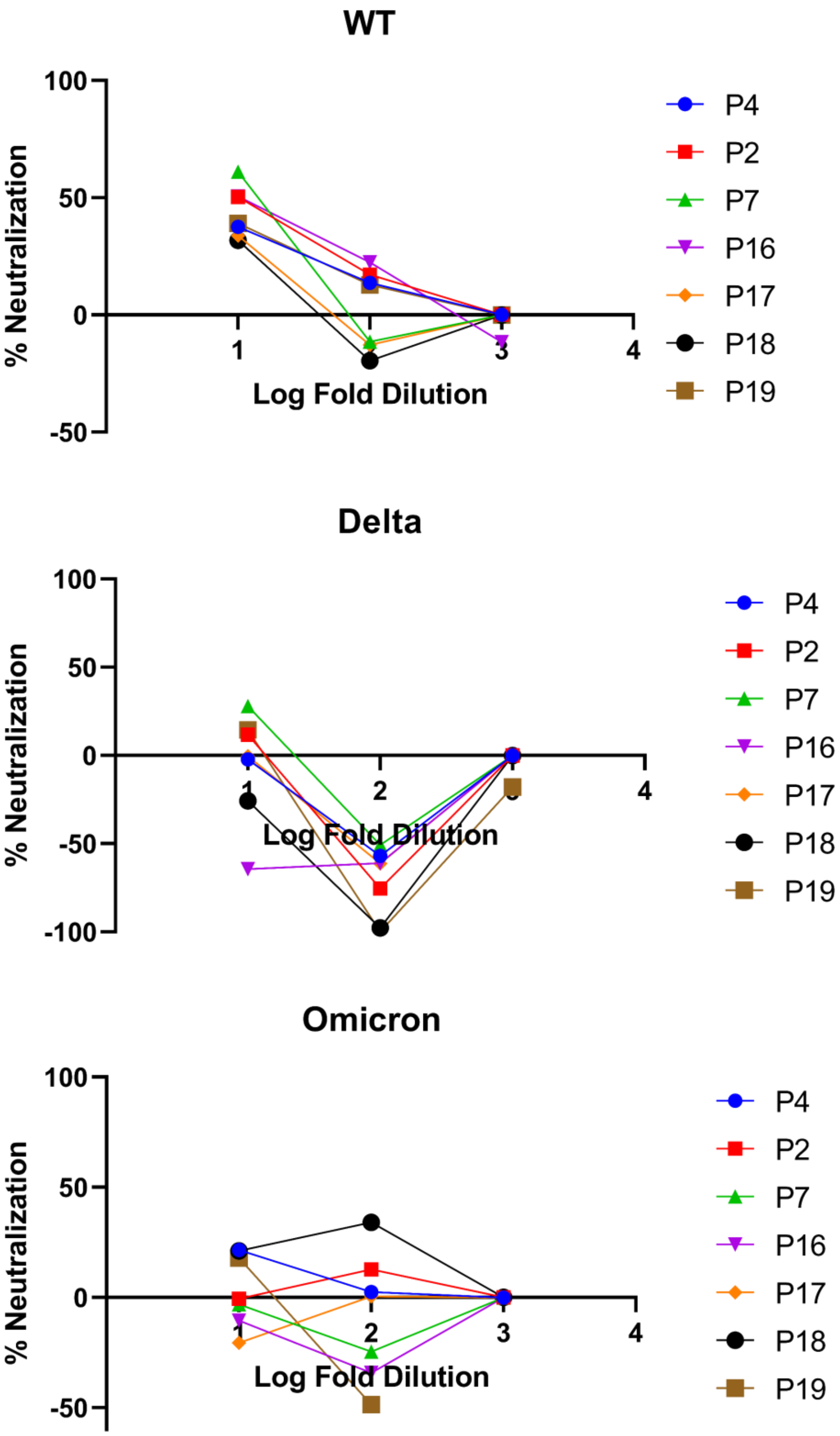


**Fig. S5. Poor neutralization of wild-type, Delta and Omicron S protein pseudotyped viruses by pre-pandemic sera.** Data show the average of (n=2) replicates.

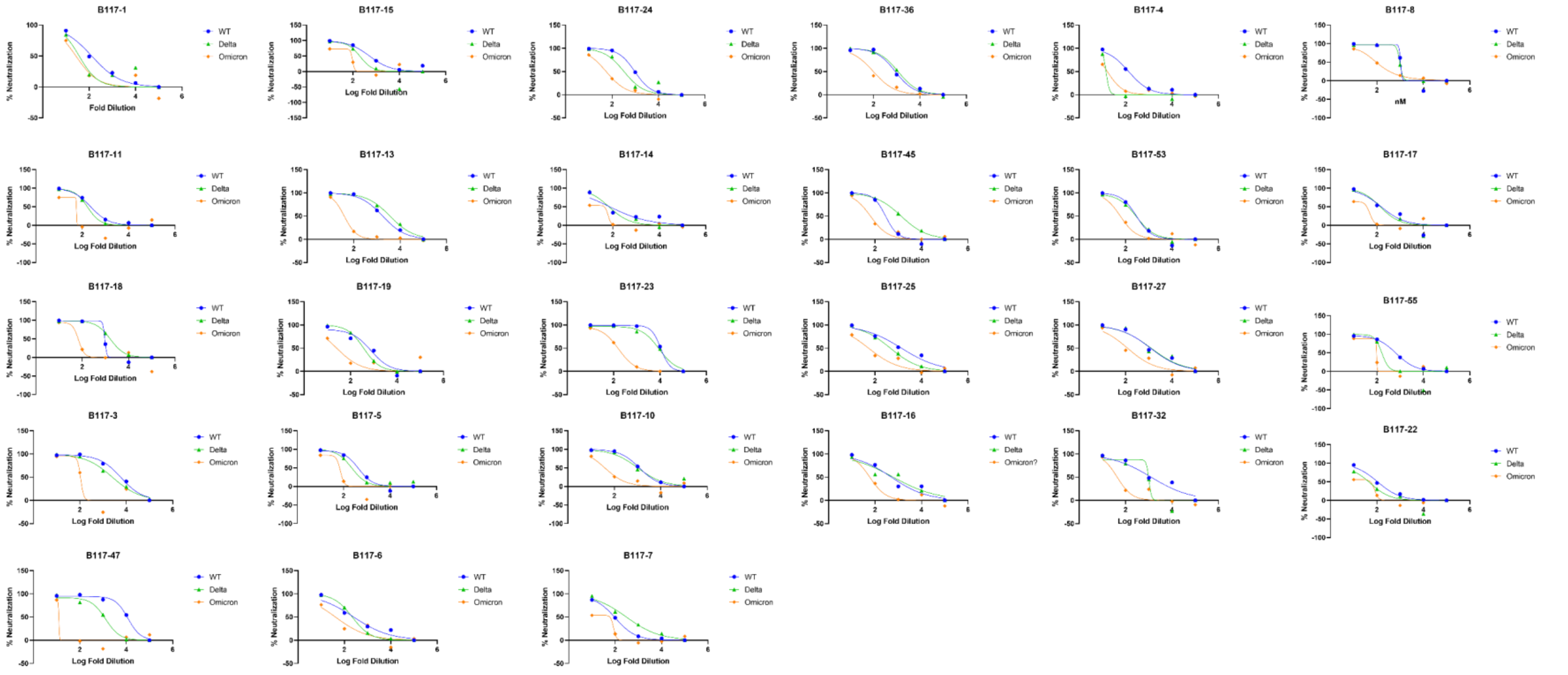


**Fig. S6. Data for the neutralization of wild-type, Delta and Omicron S protein pseudotyped viruses with convalescent sera samples from patients previously infected with the Alpha variant.** Data show the average of (n=2) replicates.

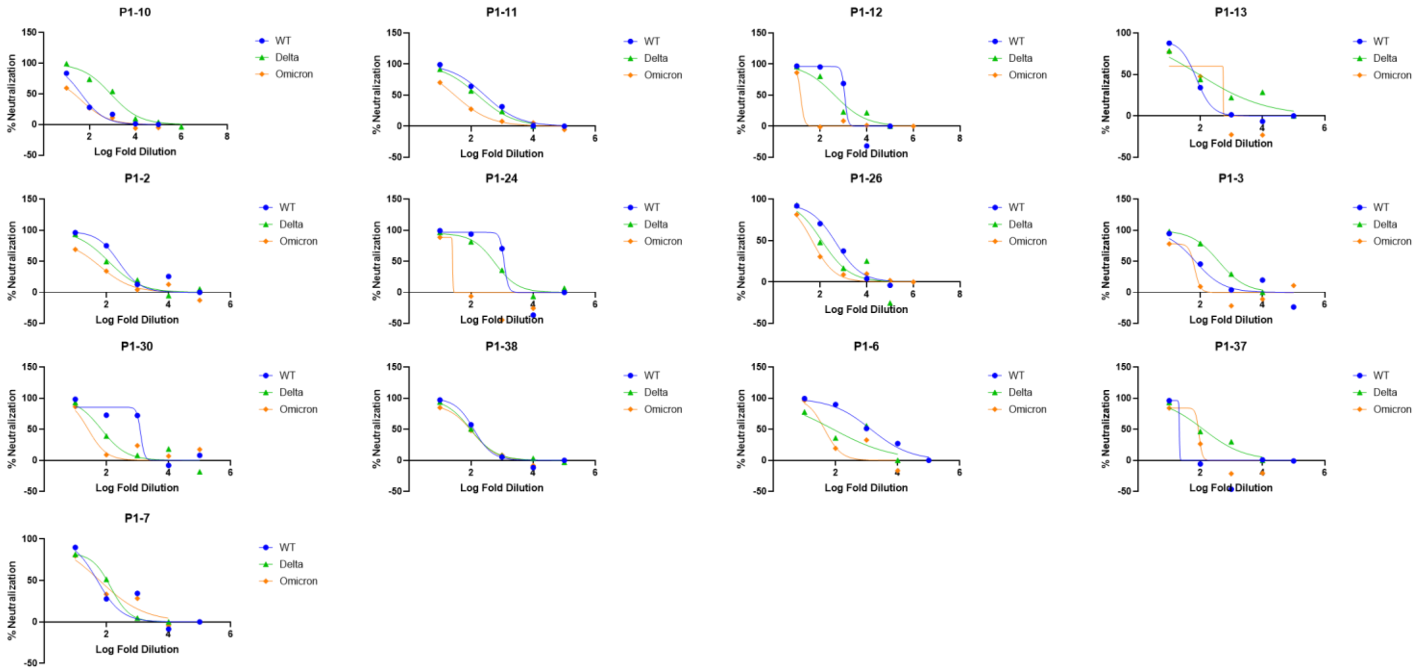


**Figure S7. Data for the neutralization of wild-type, Delta and Omicron S protein pseudotyped viruses with convalescent sera samples from patients previously infected with the Gamma variant.** Data show the average of (n=2) replicates.

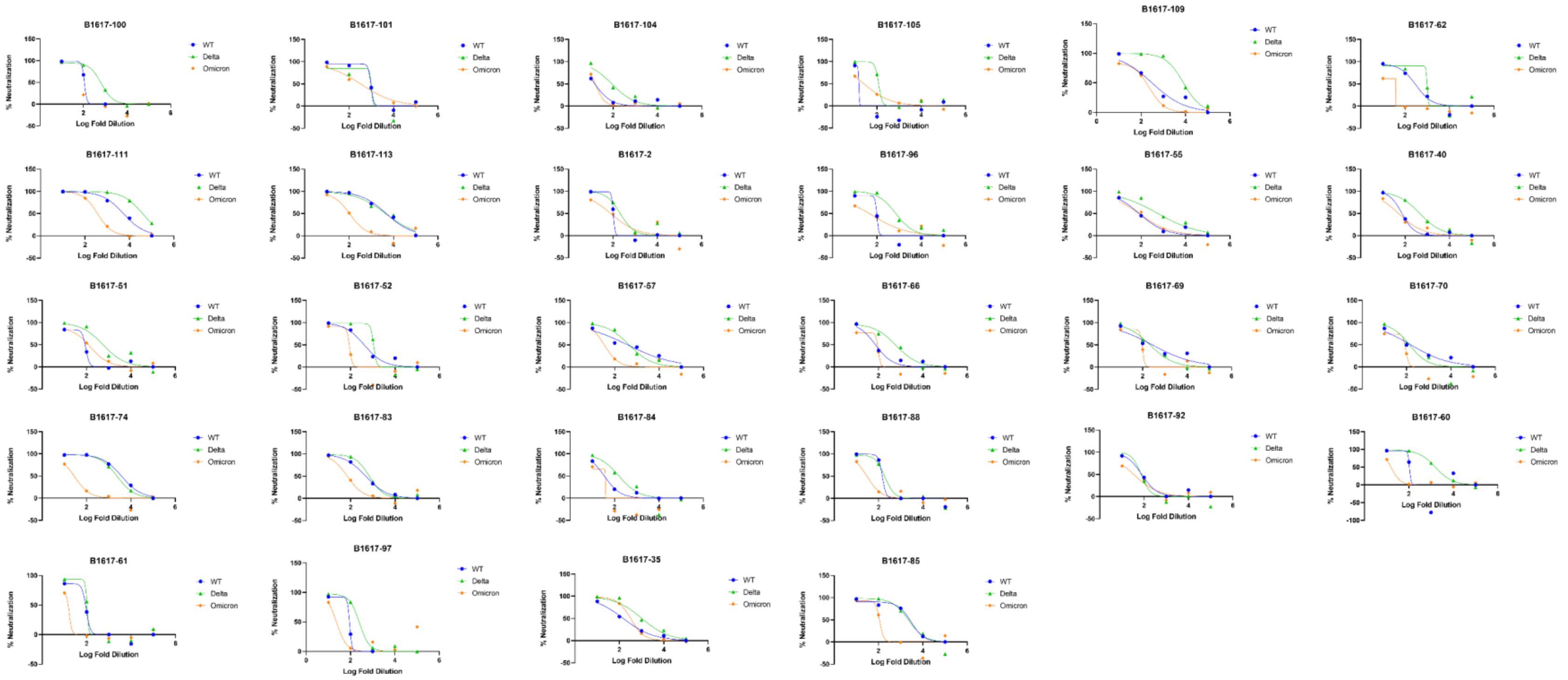


**Figure S8. Data for the neutralization of wild-type, Delta and Omicron S protein pseudotyped viruses with convalescent sera samples from patients previously infected with the Delta variant.** Data show the average of (n=2) replicates.

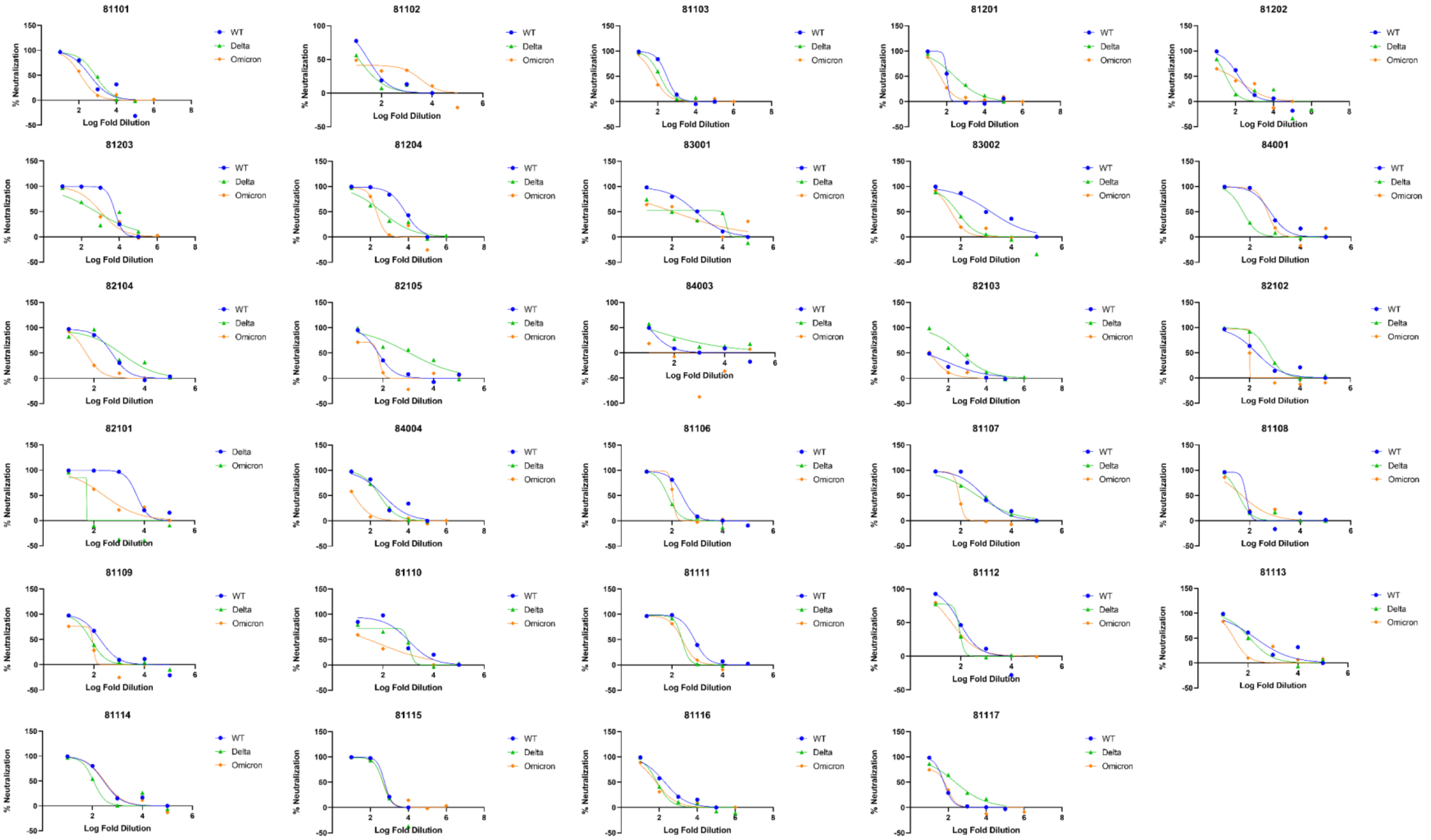


**Figure S9. Data for the neutralization of wild-type, Delta and Omicron S protein pseudotyped viruses with sera samples from non-convalescent patients who received 2 vaccine doses.** Data show the average of (n=2) replicates.

**Table S1. CryoEM data collection, processing, refinement, and validation parameters for the structures reported in this publication.**

| | S(Omicron) | S(Omicron) + ACE2 | |
|---|---|---|---|
| | | global refinement | focus refinement |
| | (EMDB 25761) | (EMDB 25759) | (EMDB 25760) |
| | (PDB 7T9L) | (PDB 7T9J) | (PDB 7T9K) |
| **Data collection** | | | |
| Microscope | Glacios | Titan Krios G4 | |
| Detector | Falcon4 | Falcon4 | |
| Voltage (kV) | 200 | 300 | |
| Nominal magnification | 190,000 | 155,000 | |
| Defocus range (μm) | -2.0 to -0.5 | -2.0 to -0.5 | |
| Physical pixel (Å) | 0.723 | 0.5 | |
| Electron dose ($e^-$/Å$^2$) | 40 | 40 | |
| Exposure rate ($e^-$/Å$^2$/sec) | 10 | 24 | |
| Format of movies | EER | EER | |
| Number of raw frames | 964 | 399 | |
| Number of movies | 2,907 | 15,768 | |
| **Data processing** | | | |
| Number of fractions | 40 | 40 | |
| Number of extracted particles | 391,755 | 1,369,874 | |
| Number of particles for final map | 236,687 | 272,266 | |
| Symmetry imposed | C1 | C1 | C1 |
| Resolution (Å) | 2.79 | 2.45 | 2.66 |
| FSC threshold | 0.143 | 0.143 | 0.143 |
| **Refinement** | | | |
| Initial model used | 7MJG | 7MJM,7MJN | 7MJN |
| Map sharpening B-factor (Å$^2$) | 72.2 | 39.2 | 82.6 |
| Composition (#) | | | |
| Atoms | 21,960 | 33,398 | 6,567 |
| Residues | 2,703 | 4,087 | 796 |
| Ligands | NAG:58 | NAG:70 | NAG:7 |
| B-factor (Å$^2$) | | | |
| Protein (min/max/mean) | 45.69/247.52/111.11 | 25.51/351.21/165.88 | 52.33/188.61/88.85 |
| Ligand (min/max/mean) | 72.19/241.41/126.73 | 54.52/304.70/152.98 | 100.53/120.00/108.29 |
| Bonds (RMSD) | | | |
| Length (Å) (# > 4σ) | 0.005 (0) | 0.004 (0) | 0.006 (0) |
| Angles (°) (# > 4σ) | 0.824 (8) | 0.842 (12) | 0.943 (4) |
| CC_mask | 0.82 | 0.79 | 0.84 |
| **Validation** | | | |
| Ramachandran plot | | | |
| Residues favored (%) | 98.22 | 97.84 | 97.22 |
| Residues disallowed (%) | 0.04 | 0.02 | 0.00 |
| Rotamer outliers (%) | 0.17 | 0.03 | 0.14 |
| Clash score | 4.49 | 3.83 | 3.89 |
| MolProbity score | 1.23 | 1.21 | 1.32 |

Table S2. Mutational effects on ACE2 binding and RBD expression as determined by Starr *et al (17)*.

| RBD Mutation | ACE2 Affinity ($\Delta log_{10}[K_{D,app}]$) | RBD Expression ($\Delta log[MFI]$) |
|---|---|---|
| G339D | 0.06 | 0.3 |
| S371L | -0.14 | -0.61 |
| S373P | -0.08 | -0.22 |
| S375F | -0.55 | -1.81 |
| K417N | -0.45 | 0.1 |
| N440K | 0.07 | -0.12 |
| G446S | -0.2 | -0.4 |
| S477N | 0.06 | 0.06 |
| T478K | 0.02 | 0.02 |
| E484A | -0.07 | -0.23 |
| Q493R | -0.09 | -0.06 |
| G496S | -0.63 | 0.12 |
| Q498R | -0.06 | -0.1 |
| N501Y | 0.24 | -0.14 |
| Y505H | -0.71 | 0.16 |

**Table S3: Summary of Patient Demographics.** The mean time from vaccine dose 2 to serum collection for the vaccinated group was 75.6 days and the mean time from infection to serum collection for the convalescent group was 29.4 days.

| Sample ID | VOC | Sample Type | Sex | Age | Days post infection | Days between first dose and serum collection | Days between second dose and serum collection | Days between first and second dose |
|---|---|---|---|---|---|---|---|---|
| 81101 | - | Vaccine - 2 Dose | M | 72 | - | 93 | 72 | 21 |
| 81102 | - | Vaccine - 2 Dose | F | 85 | - | 98 | 81 | 17 |
| 81103 | - | Vaccine - 2 Dose | M | 80 | - | 97 | 72 | 25 |
| 81106 | - | Vaccine - 2 Dose | F | 63 | - | 107 | 90 | 17 |
| 81107 | - | Vaccine - 2 Dose | F | 58 | - | 166 | 104 | 62 |
| 81108 | - | Vaccine - 2 Dose | F | 67 | - | 154 | 127 | 27 |
| 81109 | - | Vaccine - 2 Dose | F | 82 | - | 107 | 85 | 22 |
| 81110 | - | Vaccine - 2 Dose | F | 50 | - | 197 | 44 | 153 |
| 81111 | - | Vaccine - 2 Dose | F | 31 | - | 113 | 94 | 19 |
| 81112 | - | Vaccine - 2 Dose | F | 33 | - | 148 | 109 | 39 |
| 81113 | - | Vaccine - 2 Dose | F | 33 | - | 162 | 41 | 121 |
| 81114 | - | Vaccine - 2 Dose | F | 37 | - | 136 | 103 | 33 |
| 81115 | - | Vaccine - 2 Dose | M | 31 | - | 98 | 77 | 21 |
| 81116 | - | Vaccine - 2 Dose | F | 35 | - | 156 | 44 | 112 |
| 81117 | - | Vaccine - 2 Dose | F | 30 | - | 159 | 42 | 117 |
| 81201 | - | Vaccine - 2 Dose | F | 54 | - | 93 | 78 | 15 |
| 81202 | - | Vaccine - 2 Dose | F | 74 | - | 83 | 62 | 21 |
| 81203 | - | Vaccine - 2 Dose | F | 31 | - | 86 | 70 | 16 |
| 81204 | - | Vaccine - 2 Dose | F | 31 | - | 144 | 98 | 46 |
| 82101 | - | Vaccine - 2 Dose | M | 57 | - | 133 | 112 | 21 |
| 82102 | - | Vaccine - 2 Dose | M | 89 | - | 96 | 82 | 14 |
| 82103 | - | Vaccine - 2 Dose | M | 73 | - | 88 | 65 | 23 |
| 82104 | - | Vaccine - 2 Dose | M | 70 | - | 74 | 59 | 15 |
| 82105 | - | Vaccine - 2 Dose | F | 89 | - | 81 | 66 | 15 |
| 83001 | - | Vaccine - 2 Dose | M | 62 | - | 88 | 68 | 20 |
| 83002 | - | Vaccine - 2 Dose | F | 43 | - | 97 | 75 | 22 |
| 84001 | - | Vaccine - 2 Dose | F | 60 | - | 75 | 59 | 16 |
| 84003 | - | Vaccine - 2 Dose | F | 52 | - | 79 | 64 | 15 |
| 84004 | - | Vaccine - 2 Dose | M | 65 | - | 85 | 64 | 21 |
| B117-1 | B.1.1.7 | Convalescent | F | 38 | 25 | - | - | |
| B117-10 | B.1.1.7 | Convalescent | M | 53 | 15 | - | - | |
| B117-11 | B.1.1.7 | Convalescent | M | 47 | 16 | - | - | |
| B117-13 | B.1.1.7 | Convalescent | M | 59 | 17 | - | - | |
| B117-14 | B.1.1.7 | Convalescent | F | 42 | 16 | - | - | |
| B117-15 | B.1.1.7 | Convalescent | F | 37 | 29 | - | - | |
| B117-16 | B.1.1.7 | Convalescent | F | 38 | 20 | - | - | |
| B117-17 | B.1.1.7 | Convalescent | M | 49 | 19 | - | - | |
| B117-18 | B.1.1.7 | Convalescent | M | 57 | 16 | - | - | |
| B117-19 | B.1.1.7 | Convalescent | M | 34 | 54 | - | - | |
| B117-22 | B.1.1.7 | Convalescent | F | 13 | 58 | - | - | |
| B117-23 | B.1.1.7 | Convalescent | M | 50 | 17 | - | - | |
| B117-24 | B.1.1.7 | Convalescent | M | 53 | 36 | - | - | |
| B117-25 | B.1.1.7 | Convalescent | M | 30 | 21 | - | - | |
| B117-27 | B.1.1.7 | Convalescent | F | 28 | 23 | - | - | |
| B117-3 | B.1.1.7 | Convalescent | M | 55 | 15 | - | - | |
| B117-32 | B.1.1.7 | Convalescent | M | 31 | 42 | - | - | |
| B117-36 | B.1.1.7 | Convalescent | M | 50 | 21 | - | - | |
| B117-4 | B.1.1.7 | Convalescent | M | 47 | 20 | - | - | |
| B117-45 | B.1.1.7 | Convalescent | M | 35 | 27 | - | - | |
| B117-47 | B.1.1.7 | Convalescent | M | 37 | 15 | - | - | |
| B117-5 | B.1.1.7 | Convalescent | M | 18 | 22 | - | - | |
| B117-53 | B.1.1.7 | Convalescent | F | 31 | 38 | - | - | |
| B117-55 | B.1.1.7 | Convalescent | F | 68 | 59 | - | - | |
| B117-6 | B.1.1.7 | Convalescent | M | 43 | 22 | - | - | |
| B117-7 | B.1.1.7 | Convalescent | M | 16 | 16 | - | - | |
| B117-8 | B.1.1.7 | Convalescent | F | 38 | 21 | - | - | |
| B1617-100 | AY.25 | Convalescent | F | 34 | 33 | - | - | |
| B1617-101 | B.1.617.2 | Convalescent | F | 54 | 18 | - | - | |
| B1617-104 | AY.25 | Convalescent | F | 22 | 36 | - | - | |
| B1617-105 | B.1.617.2 | Convalescent | F | 28 | 32 | - | - | |
| B1617-109 | AY.27 | Convalescent | F | 28 | 46 | - | - | |
| B1617-111 | AY.27 | Convalescent | M | 67 | 21 | - | - | |
| B1617-113 | AY.27 | Convalescent | F | 26 | 19 | - | - | |
| B1617-2 | B.1.617.2 | Convalescent | F | 37 | 27 | - | - | |
| B1617-35 | B.1.617.2 | Convalescent | F | 31 | 17 | - | - | |
| B1617-40 | B.1.617.2 | Convalescent | F | 79 | 115 | - | - | |
| B1617-51 | B.1.617.2 | Convalescent | F | 25 | 57 | - | - | |
| B1617-52 | B.1.617.2 | Convalescent | F | 16 | 14 | - | - | |
| B1617-55 | B.1.617.2 | Convalescent | F | 27 | 29 | - | - | |
| B1617-57 | B.1.617.2 | Convalescent | F | 36 | 27 | - | - | |
| B1617-60 | B.1.617.2 | Convalescent | F | 60 | 22 | - | - | |
| B1617-61 | B.1.617.2 | Convalescent | M | 31 | 25 | - | - | |
| B1617-62 | B.1.617.2 | Convalescent | F | 44 | 20 | - | - | |
| B1617-66 | B.1.617.2 | Convalescent | F | 22 | 14 | - | - | |
| B1617-69 | B.1.617.2 | Convalescent | M | 28 | 40 | - | - | |
| B1617-70 | B.1.617.2 | Convalescent | F | 25 | 37 | - | - | |
| B1617-74 | B.1.617.2 | Convalescent | M | 50 | 20 | - | - | |
| B1617-83 | AY.25 | Convalescent | F | 40 | 15 | - | - | |
| B1617-84 | AY.27 | Convalescent | M | 36 | 39 | - | - | |
| B1617-85 | AY.25 | Convalescent | F | 19 | 17 | - | - | |
| B1617-88 | AY.25 | Convalescent | F | 21 | 24 | - | - | |
| B1617-92 | AY.25 | Convalescent | F | 39 | 26 | - | - | |
| B1617-96 | AY.27 | Convalescent | M | 25 | 37 | - | - | |
| B1617-97 | AY.25 | Convalescent | M | 47 | 42 | - | - | |
| P1-10 | P.1 | Convalescent | M | 38 | 23 | - | - | |
| P1-11 | P.1 | Convalescent | F | 35 | 44 | - | - | |
| P1-12 | P.1 | Convalescent | F | 24 | 37 | - | - | |
| P1-13 | P.1 | Convalescent | M | 56 | 32 | - | - | |
| P1-2 | P.1 | Convalescent | M | 35 | 21 | - | - | |
| P1-24 | P.1 | Convalescent | M | 49 | 29 | - | - | |
| P1-26 | P.1 | Convalescent | F | 21 | 18 | - | - | |
| P1-3 | P.1 | Convalescent | F | 14 | 26 | - | - | |
| P1-30 | P.1 | Convalescent | F | 55 | 25 | - | - | |
| P1-37 | P.1 | Convalescent | F | 32 | 62 | - | - | |
| P1-38 | P.1 | Convalescent | F | 27 | 60 | - | - | |
| P1-6 | P.1 | Convalescent | | 29 | 25 | - | - | |
| P1-7 | P.1 | Convalescent | M | 28 | 31 | - | - | |

## References and Notes


1. S. Elbe, G. Buckland-Merrett, Data, disease and diplomacy: GISAID's innovative contribution to global health. *Glob. Chall.* **1**, 33–46 (2017). doi:10.1002/gch2.1018 Medline

2. M. Hoffmann, H. Kleine-Weber, S. Schroeder, N. Krüger, T. Herrler, S. Erichsen, T. S. Schiergens, G. Herrler, N.-H. Wu, A. Nitsche, M. A. Müller, C. Drosten, S. Pöhlmann, SARS-CoV-2 cell entry depends on ACE2 and TMPRSS2 and is blocked by a clinically proven protease inhibitor. *Cell* **181**, 271–280.e8 (2020). doi:10.1016/j.cell.2020.02.052 Medline

3. L. Piccoli, Y.-J. Park, M. A. Tortorici, N. Czudnochowski, A. C. Walls, M. Beltramello, C. Silacci-Fregni, D. Pinto, L. E. Rosen, J. E. Bowen, O. J. Acton, S. Jaconi, B. Guarino, A. Minola, F. Zatta, N. Sprugasci, J. Bassi, A. Peter, A. De Marco, J. C. Nix, F. Mele, S. Jovic, B. F. Rodriguez, S. V. Gupta, F. Jin, G. Piumatti, G. Lo Presti, A. F. Pellanda, M. Biggiogero, M. Tarkowski, M. S. Pizzuto, E. Cameroni, C. Havenar-Daughton, M. Smithey, D. Hong, V. Lepori, E. Albanese, A. Ceschi, E. Bernasconi, L. Elzi, P. Ferrari, C. Garzoni, A. Riva, G. Snell, F. Sallusto, K. Fink, H. W. Virgin, A. Lanzavecchia, D. Corti, D. Veesler, Mapping neutralizing and immunodominant sites on the SARS-CoV-2 spike receptor-binding domain by structure-guided high-resolution serology. *Cell* **183**, 1024–1042.e21 (2020). doi:10.1016/j.cell.2020.09.037 Medline

4. C. Wei, K.-J. Shan, W. Wang, S. Zhang, Q. Huan, W. Qian, Evidence for a mouse origin of the SARS-CoV-2 Omicron variant. *J. Genet. Genomics* 10.1016/j.jgg.2021.12.003 (2021). doi:10.1016/j.jgg.2021.12.003 Medline

5. A. C. Walls, Y.-J. Park, M. A. Tortorici, A. Wall, A. T. McGuire, D. Veesler, Structure, function, and antigenicity of the SARS-CoV-2 spike glycoprotein. *Cell* **181**, 281–292.e6 (2020). doi:10.1016/j.cell.2020.02.058 Medline

6. D. Wrapp, N. Wang, K. S. Corbett, J. A. Goldsmith, C.-L. Hsieh, O. Abiona, B. S. Graham, J. S. McLellan, Cryo-EM structure of the 2019-nCoV spike in the prefusion conformation. *Science* **367**, 1260–1263 (2020). doi:10.1126/science.abb2507 Medline

7. J. Zhang, Y. Cai, T. Xiao, J. Lu, H. Peng, S. M. Sterling, R. M. Walsh Jr., S. Rits-Volloch, H. Zhu, A. N. Woosley, W. Yang, P. Sliz, B. Chen, Structural impact on SARS-CoV-2 spike protein by D614G substitution. *Science* **372**, 525–530 (2021). doi:10.1126/science.abf2303 Medline

8. X. Zhu, D. Mannar, S. S. Srivastava, A. M. Berezuk, J.-P. Demers, J. W. Saville, K. Leopold, W. Li, D. S. Dimitrov, K. S. Tuttle, S. Zhou, S. Chittori, S. Subramaniam, Cryo-electron microscopy structures of the N501Y SARS-CoV-2 spike protein in complex with ACE2 and 2 potent neutralizing antibodies. *PLOS Biol.* **19**, e3001237 (2021). doi:10.1371/journal.pbio.3001237 Medline

9. J. Zhang, T. Xiao, Y. Cai, C. L. Lavine, H. Peng, H. Zhu, K. Anand, P. Tong, A. Gautam, M. L. Mayer, R. M. Walsh Jr., S. Rits-Volloch, D. R. Wesemann, W. Yang, M. S. Seaman, J. Lu, B. Chen, Membrane fusion and immune evasion by the spike protein of SARS-CoV-2 Delta variant. *Science* **374**, 1353–1360 (2021). doi:10.1126/science.abl9463 Medline

10. S. M.-C. Gobeil, K. Janowska, S. McDowell, K. Mansouri, R. Parks, V. Stalls, M. F. Kopp, K. Manne, D. Li, K. Wiehe, K. O. Saunders, R. J. Edwards, B. Korber, B. F. Haynes, R.

Henderson, P. Acharya, Effect of natural mutations of SARS-CoV-2 on spike structure, conformation, and antigenicity. *Science* **373**, eabi6226 (2021). doi:10.1126/science.abi6226 Medline

11. K. M. Hastie, H. Li, D. Bedinger, S. L. Schendel, S. M. Dennison, K. Li, V. Rayaprolu, X. Yu, C. Mann, M. Zandonatti, R. Diaz Avalos, D. Zyla, T. Buck, S. Hui, K. Shaffer, C. Hariharan, J. Yin, E. Olmedillas, A. Enriquez, D. Parekh, M. Abraha, E. Feeney, G. Q. Horn, Y. Aldon, H. Ali, S. Aracic, R. R. Cobb, R. S. Federman, J. M. Fernandez, J. Glanville, R. Green, G. Grigoryan, A. G. Lujan Hernandez, D. D. Ho, K. A. Huang, J. Ingraham, W. Jiang, P. Kellam, C. Kim, M. Kim, H. M. Kim, C. Kong, S. J. Krebs, F. Lan, G. Lang, S. Lee, C. L. Leung, J. Liu, Y. Lu, A. MacCamy, A. T. McGuire, A. L. Palser, T. H. Rabbitts, Z. Rikhtegaran Tehrani, M. M. Sajadi, R. W. Sanders, A. K. Sato, L. Schweizer, J. Seo, B. Shen, J. L. Snitselaar, L. Stamatatos, Y. Tan, M. T. Tomic, M. J. van Gils, S. Youssef, J. Yu, T. Z. Yuan, Q. Zhang, B. Peters, G. D. Tomaras, T. Germann, E. O. Saphire, CoVIC-DB team1, Defining variant-resistant epitopes targeted by SARS-CoV-2 antibodies: A global consortium study. *Science* **374**, 472–478 (2021). doi:10.1126/science.abh2315 Medline

12. D. Mannar, J. W. Saville, X. Zhu, S. S. Srivastava, A. M. Berezuk, S. Zhou, K. S. Tuttle, A. Kim, W. Li, D. S. Dimitrov, S. Subramaniam, Structural analysis of receptor binding domain mutations in SARS-CoV-2 variants of concern that modulate ACE2 and antibody binding. *Cell Rep.* **37**, 110156 (2021). doi:10.1016/j.celrep.2021.110156 Medline

13. M. I. Barton, S. A. MacGowan, M. A. Kutuzov, O. Dushek, G. J. Barton, P. A. van der Merwe, Effects of common mutations in the SARS-CoV-2 Spike RBD and its ligand, the human ACE2 receptor on binding affinity and kinetics. *eLife* **10**, e70658 (2021). doi:10.7554/eLife.70658 Medline

14. H. Liu, Q. Zhang, P. Wei, Z. Chen, K. Aviszus, J. Yang, W. Downing, C. Jiang, B. Liang, L. Reynoso, G. P. Downey, S. K. Frankel, J. Kappler, P. Marrack, G. Zhang, The basis of a more contagious 501Y.V1 variant of SARS-CoV-2. *Cell Res.* **31**, 720–722 (2021). doi:10.1038/s41422-021-00496-8 Medline

15. F. Tian, B. Tong, L. Sun, S. Shi, B. Zheng, Z. Wang, X. Dong, P. Zheng, N501Y mutation of spike protein in SARS-CoV-2 strengthens its binding to receptor ACE2. *eLife* **10**, e69091 (2021). doi:10.7554/eLife.69091 Medline

16. C. Laffeber, K. de Koning, R. Kanaar, J. H. G. Lebbink, Experimental evidence for enhanced receptor binding by rapidly spreading SARS-CoV-2 variants. *J. Mol. Biol.* **433**, 167058 (2021). doi:10.1016/j.jmb.2021.167058 Medline

17. T. N. Starr, A. J. Greaney, S. K. Hilton, D. Ellis, K. H. D. Crawford, A. S. Dingens, M. J. Navarro, J. E. Bowen, M. A. Tortorici, A. C. Walls, N. P. King, D. Veesler, J. D. Bloom, Deep mutational scanning of SARS-CoV-2 receptor binding domain reveals constraints on folding and ACE2 binding. *Cell* **182**, 1295–1310.e20 (2020). doi:10.1016/j.cell.2020.08.012 Medline

18. J. Zahradník, S. Marciano, M. Shemesh, E. Zoler, D. Harari, J. Chiaravalli, B. Meyer, Y. Rudich, C. Li, I. Marton, O. Dym, N. Elad, M. G. Lewis, H. Andersen, M. Gagne, R. A. Seder, D. C. Douek, G. Schreiber, SARS-CoV-2 variant prediction and antiviral drug

design are enabled by RBD in vitro evolution. *Nat. Microbiol.* **6**, 1188–1198 (2021). doi:10.1038/s41564-021-00954-4 Medline

19. E. Cameroni, J. E. Bowen, L. E. Rosen, C. Saliba, S. K. Zepeda, K. Culap, D. Pinto, L. A. VanBlargan, A. De Marco, J. di Iulio, F. Zatta, H. Kaiser, J. Noack, N. Farhat, N. Czudnochowski, C. Havenar-Daughton, K. R. Sprouse, J. R. Dillen, A. E. Powell, A. Chen, C. Maher, L. Yin, D. Sun, L. Soriaga, J. Bassi, C. Silacci-Fregni, C. Gustafsson, N. M. Franko, J. Logue, N. T. Iqbal, I. Mazzitelli, J. Geffner, R. Grifantini, H. Chu, A. Gori, A. Riva, O. Giannini, A. Ceschi, P. Ferrari, P. E. Cippà, A. Franzetti-Pellanda, C. Garzoni, P. J. Halfmann, Y. Kawaoka, C. Hebner, L. A. Purcell, L. Piccoli, M. S. Pizzuto, A. C. Walls, M. S. Diamond, A. Telenti, H. W. Virgin, A. Lanzavecchia, G. Snell, D. Veesler, D. Corti, Broadly neutralizing antibodies overcome SARS-CoV-2 Omicron antigenic shift. *Nature* 10.1038/s41586-021-04386-2 (2021). doi:10.1038/s41586-021-04386-2 Medline

20. J. W. Saville, D. Mannar, X. Zhu, S. S. Srivastava, A. M. Berezuk, J.-P. Demers, S. Zhou, K. S. Tuttle, I. Sekirov, A. Kim, W. Li, D. S. Dimitrov, S. Subramaniam, Structural and biochemical rationale for enhanced spike protein fitness in Delta and Kappa SARS-CoV-2 variants. bioRxiv 2021.2009.2002.458774 [Preprint] (2021); https://doi.org/10.1101/2021.09.02.458774.

21. F. Krammer, SARS-CoV-2 vaccines in development. *Nature* **586**, 516–527 (2020). doi:10.1038/s41586-020-2798-3 Medline

22. W. Li, C. Chen, A. Drelich, D. R. Martinez, L. E. Gralinski, Z. Sun, A. Schäfer, S. S. Kulkarni, X. Liu, S. R. Leist, D. V. Zhelev, L. Zhang, Y.-J. Kim, E. C. Peterson, A. Conard, J. W. Mellors, C. K. Tseng, D. Falzarano, R. S. Baric, D. S. Dimitrov, Rapid identification of a human antibody with high prophylactic and therapeutic efficacy in three animal models of SARS-CoV-2 infection. *Proc. Natl. Acad. Sci. U.S.A.* **117**, 29832–29838 (2020). doi:10.1073/pnas.2010197117 Medline

23. W. Li, A. Schäfer, S. S. Kulkarni, X. Liu, D. R. Martinez, C. Chen, Z. Sun, S. R. Leist, A. Drelich, L. Zhang, M. L. Ura, A. Berezuk, S. Chittori, K. Leopold, D. Mannar, S. S. Srivastava, X. Zhu, E. C. Peterson, C.-T. Tseng, J. W. Mellors, D. Falzarano, S. Subramaniam, R. S. Baric, D. S. Dimitrov, High potency of a bivalent human $V_H$ domain in SARS-CoV-2 animal models. *Cell* **183**, 429–441.e16 (2020). doi:10.1016/j.cell.2020.09.007 Medline

24. M. A. Tortorici, M. Beltramello, F. A. Lempp, D. Pinto, H. V. Dang, L. E. Rosen, M. McCallum, J. Bowen, A. Minola, S. Jaconi, F. Zatta, A. De Marco, B. Guarino, S. Bianchi, E. J. Lauron, H. Tucker, J. Zhou, A. Peter, C. Havenar-Daughton, J. A. Wojcechowskyj, J. B. Case, R. E. Chen, H. Kaiser, M. Montiel-Ruiz, M. Meury, N. Czudnochowski, R. Spreafico, J. Dillen, C. Ng, N. Sprugasci, K. Culap, F. Benigni, R. Abdelnabi, S. C. Foo, M. A. Schmid, E. Cameroni, A. Riva, A. Gabrieli, M. Galli, M. S. Pizzuto, J. Neyts, M. S. Diamond, H. W. Virgin, G. Snell, D. Corti, K. Fink, D. Veesler, Ultrapotent human antibodies protect against SARS-CoV-2 challenge via multiple mechanisms. *Science* **370**, 950–957 (2020). doi:10.1126/science.abe3354 Medline

25. D. Pinto, Y.-J. Park, M. Beltramello, A. C. Walls, M. A. Tortorici, S. Bianchi, S. Jaconi, K. Culap, F. Zatta, A. De Marco, A. Peter, B. Guarino, R. Spreafico, E. Cameroni, J. B.

Case, R. E. Chen, C. Havenar-Daughton, G. Snell, A. Telenti, H. W. Virgin, A. Lanzavecchia, M. S. Diamond, K. Fink, D. Veesler, D. Corti, Cross-neutralization of SARS-CoV-2 by a human monoclonal SARS-CoV antibody. *Nature* **583**, 290–295 (2020). doi:10.1038/s41586-020-2349-y Medline

26. L. Liu, P. Wang, M. S. Nair, J. Yu, M. Rapp, Q. Wang, Y. Luo, J. F.-W. Chan, V. Sahi, A. Figueroa, X. V. Guo, G. Cerutti, J. Bimela, J. Gorman, T. Zhou, Z. Chen, K.-Y. Yuen, P. D. Kwong, J. G. Sodroski, M. T. Yin, Z. Sheng, Y. Huang, L. Shapiro, D. D. Ho, Potent neutralizing antibodies against multiple epitopes on SARS-CoV-2 spike. *Nature* **584**, 450–456 (2020). doi:10.1038/s41586-020-2571-7 Medline

27. X. Chi, R. Yan, J. Zhang, G. Zhang, Y. Zhang, M. Hao, Z. Zhang, P. Fan, Y. Dong, Y. Yang, Z. Chen, Y. Guo, J. Zhang, Y. Li, X. Song, Y. Chen, L. Xia, L. Fu, L. Hou, J. Xu, C. Yu, J. Li, Q. Zhou, W. Chen, A neutralizing human antibody binds to the N-terminal domain of the Spike protein of SARS-CoV-2. *Science* **369**, 650–655 (2020). doi:10.1126/science.abc6952 Medline

28. D. Mannar, J. W. Saville, Z. Sun, X. Zhu, M. M. Marti, S. S. Srivastava, A. M. Berezuk, S. Zhou, K. S. Tuttle, M. D. Sobolewski, A. Kim, B. R. Treat, P. M. Da Silva Castanha, J. L. Jacobs, S. M. Barratt-Boyes, J. W. Mellors, D. S. Dimitrov, W. Li, S. Subramaniam, Emerging SARS-CoV-2 variants of concern: Spike protein mutational analysis and epitope for broad neutralization. bioRxiv 2021.12.17.473178 [Preprint] (2021); https://doi.org/10.1101/2021.12.17.473178.

29. L. VanBlargan, J. M. Errico, P. J. Halfmann, S. J. Zost, J. E. Crowe Jr., L. A. Purcell, Y. Kawaoka, D. Corti, D. H. Fremont, M. S. Diamond, An infectious SARS-CoV-2 B.1.1.529 Omicron virus escapes neutralization by several therapeutic monoclonal antibodies. bioRxiv 2021.2012.2015.472828 [Preprint] (2021); https://doi.org/10.1101/2021.12.15.472828.

30. A. Cathcart, C. Havenar-Daughton, F. A. Lempp, D. Ma, M. A. Schmid, M. L. Agostini, B. Guarino, J. Diiulio, L. E. Rosen, H. Tucker, J. Dillen, S. Subramanian, B. Sloan, S. Bianchi, D. Pinto, C. Saliba, K. Culap, J. A. Wojcechowskyj, J. Noack, J. Zhou, H. Kaiser, A. Chase, M. Montiel-Ruiz, E. Dellota Jr., A. Park, R. Spreafico, A. Sahakyan, E. J. Lauron, N. Czudnochowski, E. Cameroni, S. Ledoux, A. Werts, C. Colas, L. Soriaga, A. Telenti, L. A. Purcell, S. Hwang, G. Snell, H. W. Virgin, D. Corti, C. M. Hebner, The dual function monoclonal antibodies VIR-7831 and VIR-7832 demonstrate potent in vitro and in vivo activity against SARS-CoV-2. bioRxiv 2021.03.09.434607 [Preprint] (2021); https://doi.org/10.1101/2021.03.09.434607.

31. M. McCallum, N. Czudnochowski, L. E. Rosen, S. K. Zepeda, J. E. Bowen, J. R. Dillen, A. E. Powell, T. I. Croll, J. Nix, H. W. Virgin, D. Corti, G. Snell, D. Veesler, Structural basis of SARS-CoV-2 Omicron immune evasion and receptor engagement. bioRxiv 2021.2012.2028.474380 [Preprint] (2021); https://doi.org/10.1101/2021.12.28.474380.

32. D. Planas, N. Saunders, P. Maes, F. Guivel-Benhassine, C. Planchais, J. Buchrieser, W. H. Bolland, F. Porrot, I. Staropoli, F. Lemoine, H. Péré, D. Veyer, J. Puech, J. Rodary, G. Baele, S. Dellicour, J. Raymenants, S. Gorissen, C. Geenen, B. Vanmechelen, T. Wawina-Bokalanga, J. Martí-Carreras, L. Cuypers, A. Sève, L. Hocqueloux, T. Prazuck, F. Rey, E. Simon-Loriere, T. Bruel, H. Mouquet, E. André, O. Schwartz, Considerable

escape of SARS-CoV-2 Omicron to antibody neutralization. *Nature* 10.1038/s41586-021-04389-z (2021). doi:10.1038/s41586-021-04389-z Medline

33. A. Rössler, L. Riepler, D. Bante, D. v. Laer, J. Kimpel, SARS-CoV-2 B.1.1.529 variant (Omicron) evades neutralization by sera from vaccinated and convalescent individuals. medRxiv 2021.2012.2008.21267491 [Preprint] (2021); https://doi.org/10.1101/2021.12.08.21267491.

34. L. Liu, S. Iketani, Y. Guo, J. F.-W. Chan, M. Wang, L. Liu, Y. Luo, H. Chu, Y. Huang, M. S. Nair, J. Yu, K. K.-H. Chik, T. T.-T. Yuen, C. Yoon, K. K.-W. To, H. Chen, M. T. Yin, M. E. Sobieszczyk, Y. Huang, H. H. Wang, Z. Sheng, K.-Y. Yuen, D. D. Ho, Striking antibody evasion manifested by the Omicron variant of SARS-CoV-2. *Nature* 10.1038/s41586-021-04388-0 (2021). doi:10.1038/s41586-021-04388-0 Medline

35. A. Punjani, J. L. Rubinstein, D. J. Fleet, M. A. Brubaker, cryoSPARC: Algorithms for rapid unsupervised cryo-EM structure determination. *Nat. Methods* **14**, 290–296 (2017). doi:10.1038/nmeth.4169 Medline

36. E. F. Pettersen, T. D. Goddard, C. C. Huang, G. S. Couch, D. M. Greenblatt, E. C. Meng, T. E. Ferrin, UCSF Chimera—A visualization system for exploratory research and analysis. *J. Comput. Chem.* **25**, 1605–1612 (2004). doi:10.1002/jcc.20084 Medline

37. P. Emsley, B. Lohkamp, W. G. Scott, K. Cowtan, Features and development of Coot. *Acta Crystallogr. D Biol. Crystallogr.* **66**, 486–501 (2010). doi:10.1107/S0907444910007493 Medline

38. D. Liebschner, P. V. Afonine, M. L. Baker, G. Bunkóczi, V. B. Chen, T. I. Croll, B. Hintze, L.-W. Hung, S. Jain, A. J. McCoy, N. W. Moriarty, R. D. Oeffner, B. K. Poon, M. G. Prisant, R. J. Read, J. S. Richardson, D. C. Richardson, M. D. Sammito, O. V. Sobolev, D. H. Stockwell, T. C. Terwilliger, A. G. Urzhumtsev, L. L. Videau, C. J. Williams, P. D. Adams, Macromolecular structure determination using x-rays, neutrons and electrons: Recent developments in Phenix. *Acta Crystallogr. D Struct. Biol.* **75**, 861–877 (2019). doi:10.1107/S2059798319011471 Medline

39. T. D. Goddard, C. C. Huang, E. C. Meng, E. F. Pettersen, G. S. Couch, J. H. Morris, T. E. Ferrin, UCSF ChimeraX: Meeting modern challenges in visualization and analysis. *Protein Sci.* **27**, 14–25 (2018). doi:10.1002/pro.3235 Medline
